# Dual targeted gene delivery strategy mediated by GalNAc-modified lipid nanoparticles enhances liver regeneration through specific knockdown of MKK4

**DOI:** 10.1016/j.mtbio.2026.103059

**Published:** 2026-03-24

**Authors:** Xiao-Pei Zhai, Jie-Hua Xing, Li-Shuang Hou, Tang-Rui Zhang, Wei He, Li-She Gan, Si-Yuan Zhou, Bang-Le Zhang

**Affiliations:** a Department of Pharmaceutics, School of Pharmacy, Fourth Military Medical University, Xi'an, 710032, China; b Key Laboratory of Gastrointestinal Pharmacology of the State Administration of Traditional Chinese Medicine, Fourth Military Medical University, Xi'an, 10032, China; c Department of Chemistry, School of Pharmacy, Fourth Military Medical University, Xi'an, 710032, China; d School of Pharmaceutical Science, Zhejiang Chinese Medical University, Hangzhou, 311402, China

**Keywords:** Liver regeneration, Acute-on-chronic liver failure, Targeted delivery system, Lipid nanoparticles, Gene therapy

## Abstract

Acute and chronic liver diseases are often related to the disorder of liver regeneration. However, there is no drug specifically approved for promoting liver regeneration especially in acute-on-chronic liver failure (ACLF). According to recent studies, inhibition of mitogen-activated protein kinase kinase 4 (MKK4) protein is critical for promoting hepatocyte proliferation and liver regeneration. However, MKK4 is widely present in various parts of the body as well with the similar binding pocket as MKK7 for small molecular inhibitors, and non-specific inhibition of MKK4 expression may pose an off-target risk in clinical practice. Therefore, MKK4-siRNA (siMKK4) was designed and optimized as a therapeutic gene for specific knockdown of MKK4 and avoid the off-target binding to mitogen-activated protein kinase kinase 7 (MKK7). Moreover, N-acetylgalactosamine (GalNAc)-modified lipid nanoparticles (LNPs) were used as gene delivery carriers to construct a dual targeted gene therapy system GalNAc-LNP-siMKK4 with liver tropism and active targeting to hepatocyte. GalNAc-LNP-siMKK4 can be efficiently constructed by the reverse phase evaporation method, with uniform particle size, good stability, biocompatibility, hepatocyte targeting ability, and high gene silence effect on the expression of MKK4. *In vitro* and *in vivo* experiments demonstrated that GalNAc-LNP-siMKK4 had good efficacy of gene therapy on promoting liver regeneration, reducing hepatocytes apoptosis, and promoting liver function recovery. The constructed hepatocyte-targeted gene therapy system GalNAc-LNP-siMKK4 could hold promise for treating ACLF based on reducing protein expression of MKK4 to promote hepatocytes proliferation specifically mediated by the targeting moiety of GalNAc. GalNAc-LNP-siMKK4 has targeting ability to deliver therapeutic genes to liver and hepatocytes, achieving a highly efficient gene therapy for promotion of liver regeneration and providing new therapeutic strategies for ACLF.

## Highlights

• Provide a promising targeted gene therapy strategy to enhance the liver regeneration.
• Combine liver tropism and active targeting for superior hepatocyte-targeted specific protein knockdown enabled by MKK4-siRNA.
• Construct the therapy system GalNAc-LNP-siMKK4A for the treatment of acute-on-chronic liver failure.

## Graphical abstract

**Figure:**
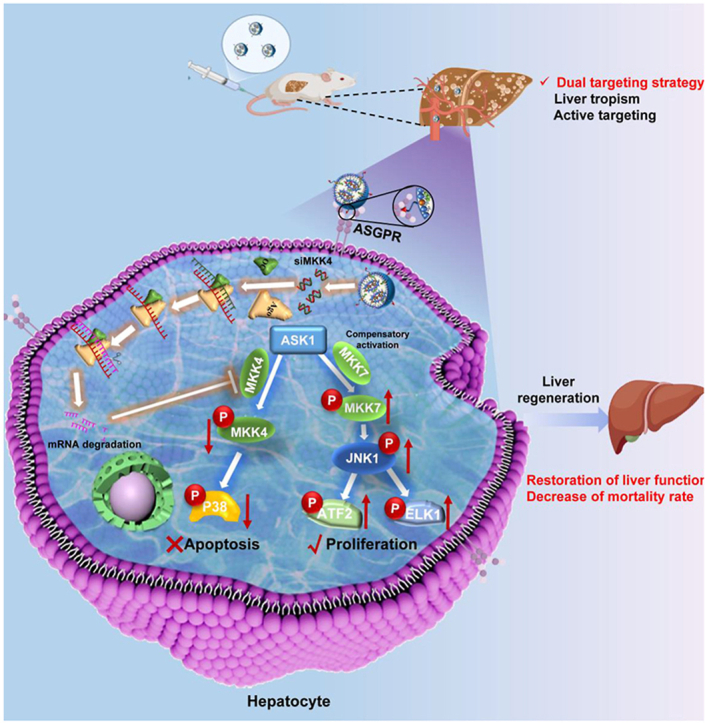

## 1 Introduction

Acute and chronic liver diseases, including alcoholic liver injury, drug-induced liver injury, liver fibrosis, hepatitis, cirrhosis, fatty liver, and liver cancer, have become a growing global burden, and are closely related to impaired liver regeneration [1,2]. Liver exhibits extraordinary regenerative potential [3,4], but acute or chronic injury can weaken this capacity, leading to liver dysfunction and liver failure [5]. For the liver diseases patients, particularly underlying chronic diseases situation, liver resection often impairs their liver regeneration [6,7], increasing the risk of acute-on-chronic liver failure (ACLF) after partial hepatectomy (PHx) with the mortality rate of 25% - 30% [[8], [9], [10]]. The prognosis of surgical resection of the liver, treatment of ACLF, and congenital liver metabolic diseases all depend on liver regeneration. Therefore, promoting liver regeneration ability is crucial for controlling liver injury, and intervention strategies are urgently needed to regulate liver regeneration.

Liver transplantation is currently recognized as the ultimate clinical option for ACLF, which can replace damaged liver [11]. However, due to the shortage of donors, significant surgical trauma, and increased risk of infection caused by long-term use of immunosuppressants, it is difficult to widely apply in clinical practice [12]. The artificial liver support system can temporarily replace the liver to perform metabolic and detoxification functions, buying time for liver regeneration or liver transplantation [13]. However, it can only alleviate symptoms and cannot repair the damaged liver regeneration ability. Moreover, there are complications such as decreased clotting, haemodynamic instability, electrolyte disturbances and infection [14]. In terms of drug therapy, currently in clinical interventions rely on liver protective drugs that can alleviate postoperative liver pathological damage through anti-inflammatory, detoxifying, and choleretic but cannot directly promote liver regeneration [[15], [16], [17], [18]]. So far, there is no drug specifically approved for promoting liver regeneration. Hepatocytes, accounting for 69% of liver cells, are the primary functional cells of the liver and play a crucial role in the process of liver injury and regeneration [2,19]. Therefore, identifying therapies that promote the hepatocytes proliferation is crucial for enhancing residual liver regeneration ability and improving liver function.

Mitogen-activated protein kinase kinase 4 (MKK4), a mitogen-activated protein 2 (MAP2) kinase in the stress-activated protein kinase (SAPK)/mitogen-activated protein kinase (MAPK) signaling network, regulates cell proliferation and differentiation [[20], [21], [22]]. There is increasing evidence recently that inhibition of MKK4 can promote hepatocyte proliferation and SAPK signaling pathway [23,24] and promoting downstream pro-regenerative transcription mediated by activating transcription factor 2 (ATF2) and ETS transcription factor ELK1 (ELK1) [25,26]. Additionally, inhibition of MKK4 can stabilize hepatocytes during injury and reduce apoptosis by downregulating p38 mitogen-activated protein kinase (p38) [[27], [28], [29]]. However, MKK4 is widely expressed *in vivo* and non-specific inhibition may increase the off-target risk in clinical practice [30]. In recent years, small molecular inhibitors for MKK4 have been gradually developed, but there are target specificity issues due to the similar binding pocket between MKK4 and mitogen-activated protein kinase kinase 7 (MKK7), which also leads to off-target binding to MKK7 [[31], [32], [33], [34]].

Compared with small molecular inhibitors, small interfering RNA (siRNA) has received widespread attention by precise targeted silencing of target genes/proteins [35]. Currently, siRNA has been widely used in the treatment of liver disease. Lv et al. used siRNA targeting silent hypoxia-inducible factor-1α to treat metabolic dysfunction-associated fatty liver disease [36]. Huang et al. used siRNA to silence acyl-CoA synthase long chain family member 3 (ACSL3) to regulate lipid metabolism, inhibiting the growth and metastasis of hepatocellular carcinoma [37]. The key to achieving ideal gene therapeutic effect of siRNA is effective delivery of them into the target cells, so selecting a suitable gene delivery vector is particularly important [38,39]. The currently widely used gene delivery vectors mainly include viral vectors and non-viral vectors [[40], [41], [42], [43]]. Although viral vectors have high transfection efficiency, they still have many drawbacks that limit their application, such as complex preparation processes, immunogenicity, and lack of cellular targeting ability [44,45]. In contrast, non-viral vectors (liposomes, lipid nanoparticles (LNPs), etc) have lower toxicity, lower immune response, and are easy to be prepared and modified by targeting moiety [46,47]. Therefore, more and more non-viral vectors are being developed and applied [[48], [49], [50]]. LNPs are typically composed of cationic lipids, auxiliary lipids, and cholesterol [[51], [52], [53]], which can protect siRNA from degradation *in vivo*. LNPs have been developed for the treatment of liver diseases due to their good liver targeting properties [54,55]. Guo et al. constructed an ionizable lipid nanoparticle (iLNPs) system for delivering siRNA therapy for liver cancer [56]. Although LNPs have good liver tropism, they cannot accurately target liver parenchymal cells and may result in poor gene silencing outcome and off-target side effects. Therefore, increasing its targeting ability towards the effector cells of hepatocytes can solve this problem.

The asialoglycoprotein receptor (ASGPR) is a highly expressed specific receptor on hepatocytes [57,58], and the molecular moiety, such as asialoglycoprotein, galactose, galactosamine, N-acetylgalactosamine (GalNAc), etc. have high affinity to ASGPR. Recently, many approved drugs, such as Givosiran and Vutrisiran, utilize GalNAc for the hepatocyte-targeted delivery of siRNA [59]. Therefore, LNPs modified by targeting moiety-GalNAc are expected to efficiently target hepatocytes and be developed as gene delivery vector for targeted gene therapy of MKK4-siRNA (siMKK4).

**Fig. 1:**
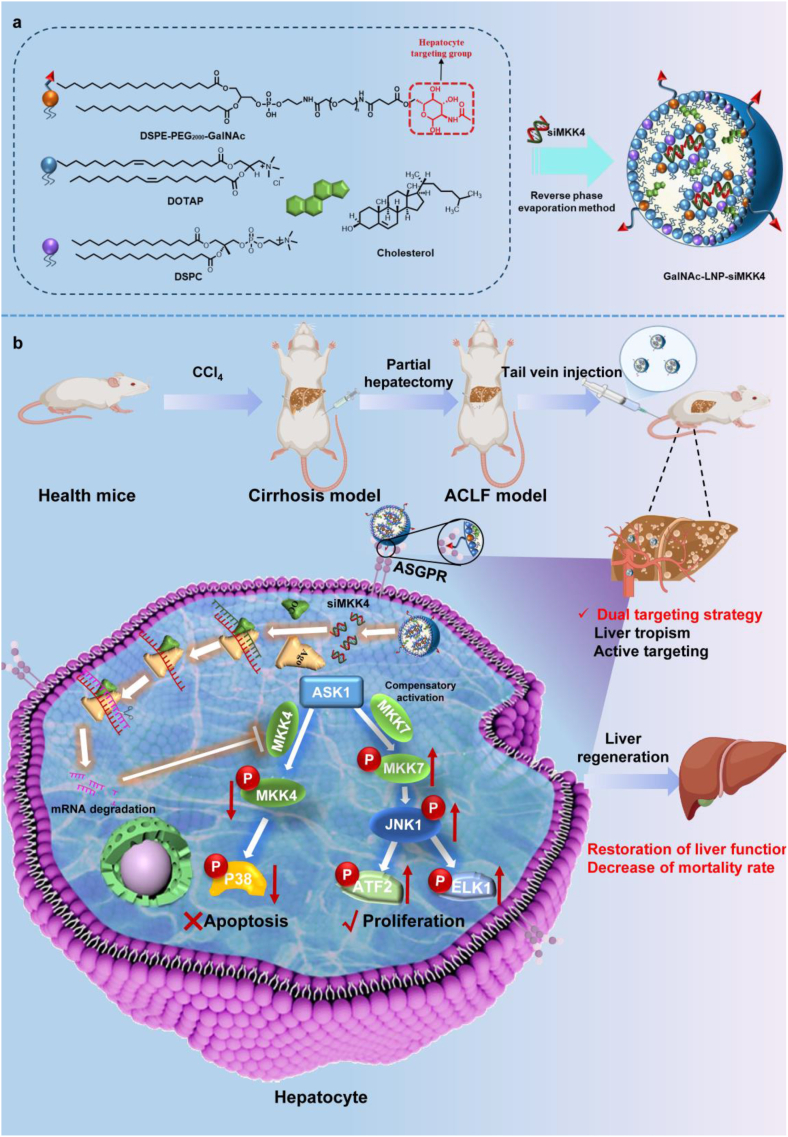
Schematic illustration of preparation and application of hepatocyte-targeted gene therapy system GalNAc-LNP-siMKK4 for the treatment of ACLF by enhancing liver regeneration. (a) Preparation of GalNAc-LNP-siMKK4 using DOTAP, DSPC, cholesterol, GalNAc-PEG_2000_-DSPE, and siMKK4. (b) Mechanism and effect of GalNAc-LNP-siMKK4 for liver and hepatocyte targeting in the treatment of ACLF.

This project aims to design a dual targeted gene therapy system GalNAc-LNP-siMKK4, which achieves precise targeting of hepatocyte by combining the liver tropism of LNPs and modifying the targeting moiety of GalNAc on LNPs through a dual targeting mechanism (Fig. 1). The sequences of siMKK4 were designed, screened and optimized as the therapeutic gene for promoting liver regeneration, and GalNAc-modified LNPs were used as gene delivery carriers to construct the gene therapy system GalNAc-LNP-siMKK4 by the reverse phase evaporation method (Fig. 1a). After injection *in vivo*, the constructed GalNAc-LNP-siMKK4 can effectively distribute in the liver with the help of liver tropism and subsequently enter hepatocytes through the specific targeting group GalNAc to ASGPR receptors on the surface of hepatocytes, showing precise gene silence effect on the expression of MKK4, thereby downregulating the MKK4-p38 pathway (apoptotic pathway), and upregulating the MKK7-JNK pathway (cell proliferation), leading to liver regeneration, liver function recovery, and reduced mortality rate (Fig. 1b). Apoptosis signal-regulating kinase 1 (ASK1) is a kinase of both MKK4 and MKK7. Inhibiting the expression of MKK4 will lead to compensatory activation of MKK7 by ASK1 through phosphorylation [[23], [24], [25], [26]]. The activated MKK7 subsequently activates JNK1, which then further activates the downstream transcription factors ATF2 and ELK1, initiating the gene expression of cell proliferation. Concurrently, reduced expression of MKK4 can attenuate the activation of p38, thereby diminishing p38-mediated pro-apoptosis and growth-suppress [[27], [28], [29]].

## 2 Materials and methods

### 2.1 Materials and reagents

DOTAP, DSPC, cholesterol, and GalNAc were purchased from Aladdin (Shanghai, China). siMKK4, siNC, and siNC-FAM were purchased from GenePharma (Shanghai, China). Agarose was purchased from Bioweste (Spain). TAE buffer (50 × ) was purchased from Shaanxi Zhhcbio Company (Xi'an, China). SerRed nucleic acid dye, 4% paraformaldehyde, Alexa Fluor 488 and Alexa Fluor 594 were purchased from Servicebio Company (Wuhan, China). Ribonuclease (RNase) was purchased from Takara Biotechnology Company (Beijing, China). Ethylene diamine tetraacetic acid (EDTA), sodium salt of dextran sulfate, DMSO, Tween 20, and Triton X-100 were purchased from Macklin Reagent Company (Shanghai, China). AM/PI and Crystal Violet were purchased from Biosharp (Hefei, China). Methylthiazolyldiphenyl-tetrazolium bromide (MTT) was purchased from Beyotime Biotechnology (Shanghai, China). DAPI was purchased from KeHao Biological Technology (Xi'an, China). CCl_4_ was purchased from Chemreagent (Tianjin, China). HNF-4α antibody and GAPDH antibody were purchased from Abcam (Cambridge, UK). MKK4 antibody and p-ATF2 antibody were purchased from Proteintech (Wuhan, China). p-MKK4 antibody was purchased from ABclonal (Wuhan, China). p-p38 antibody, p-ELK1 antibody and MKK7 antibody were purchased from Affinity (Jiangsu, China). p-MKK7 antibody was purchased from Bioss (Beijing, China). p-JNK1 antibody was purchased from Cell Signaling Technology (Massachusetts, USA). Horseradish peroxidase (HRP)-conjugated goat anti-rabbit immunoglobulin G (IgG) secondary antibody was purchased from Zhuangzhi Biotech, Inc (Xi'an, China). Radioimmunoprecipitation assay (RIPA) and fetal bovine serum (FBS) were purchased from Heyuan Liji Biotechnology (Shanghai, China). Iodixanol injection was purchased from GE Healthcare AS (Shanghai, China). DMEM/nutrient mixture F-12 (F-12) was purchased from Procell (Wuhai, China). Dehydrocorydaline chloride and SP600125 were purchased from MedChemExpress (New Jersey, United States).

### 2.2 Cells and animals

Alpha mouse liver 12 (AML-12) cells were purchased from HaiXing Biosciences Co., Ltd (Suzhou, China). Kunming mice (5-6 weeks, 25-30 g in body weight, male) were provided by the Experimental Animal Center of the Fourth Military Medical University. All animal experiments were approved by the Animal Ethics Committee of the Fourth Military Medical University (NO: 20,250,099).

### 2.3 Synthesis and characterization of GalNAc-PEG_2000_-DSPE

GalNAc (20 mg), succinic anhydride (9 mg, 1.0 eq), and DMAP (5.5 mg, 0.5 eq) were dissolved in 2 mL pyridine. The reaction was carried out at 40 °C for 2 h. The reaction solution was concentrated by vacuum distillation, precipitated with a large amount of ice ether, and vacuum dried to obtain GalNAc-COOH. Then, DSPE-PEG_2000_-NH_2_ (100 mg, 0.4 eq), GalNAc-COOH (86.7 mg, 3.0 eq), PyBOP (140.5 mg, 3.0 eq), and triethylamine (45.6 mg, 5.0 eq) were dissolved in 3 mL DMF and stirred at room temperature for 0.5 h. The reaction solution was concentrated by vacuum distillation and dissolved in ethanol. The solution was transferred to a dialysis bag (molecular weight cutoff of 1000 Da) and dialyzed in pure water for 24 h. The collected dialysate was freeze-dried to obtain DSPE-PEG_2000_-GalNAc.

### 2.4 Design and screen the gene sequences of siMKK4

AML-12 cells were seeded into a 24-well plate (1 × 10^5^/well) and cultured for 12 h until 70% fusion was achieved for gene transfection. siMKK4-1, siMKK4-2, and siMKK4-3 was mixed and incubated with lipofectamine 2000 respectively for 20 min for future use. The cell culture medium was replaced with the above mixture of siRNA and lipofectamine 2000 with a final concentration of 50 nM siRNA per well. The 24-well plate was placed in a CO_2_ incubator for 6 h, and then the cell culture medium was replaced with serum containing medium. After incubated for 24 h, quantitative reverse-transcription polymerase chain reaction (qRT-PCR) was performed by high yield real-time fluorescence quantitative PCR instrument (Thermo Fisher Company, QuantStudio, Singapore).

### 2.5 Cell culture

AML-12 cells were cultured in DMEM/F12 containing 10% FBS, 1% ITS, 1% 40 ng/mL dexamethasone, and 1% penicillin/streptomycin. Throughout the entire cultivation process, all cells were cultured in a humidified incubator with 5% CO_2_ at 37 °C.

### 2.6 Total RNA isolation and quantitative reverse-transcription polymerase chain reaction (qRT-PCR)

Total RNA was extracted using the Trizol reagent. Then, cDNA was generated by fast cDNA First Chain Synthesis Kit, qRT-PCR (Thermo Fisher Company, QuantStudio, Singapore) was performed by using a superReal Fluorescence quantification kit. The used primer sequences are listed in Table S1.

### 2.7 N/P screening of LNP and siMKK4

Preparation of agarose gel: Agarose powder (500 mg) and TAE buffer solution (50 mL) were placed in a 100 mL conical flask, and heated in a microwave oven for 1 min to melt all agarose. SerRed nucleic acid dye (6 μL) was added and shaken until fully mixed. The agarose solution was poured into the slot in the glass plate with comb, and placed at room temperature for 20 min. After the gel was completely solidified, the comb was gently pulled out vertically for standby.

Gene loading capacity assessment: DOTAP, DSPC, cholesterol, and GalNAc-PEG_2000_-DSPE were dissolved in anhydrous ethanol at concentrations of 2 mg/mL, 2 mg/mL, 2 mg/mL, and 0.5 mg/mL, respectively. siMKK4 was dissolved in DEPC water at a concentration of 0.1 μg/mL. According to the concentrations required in the experiment, different volumes of carrier solution and siMKK4 solution were measured. The siMKK4 solution was added dropwise to the carrier solution, sonicated for 20 min, and left at room temperature for 20 min. The ethanol was removed by rotary evaporation to obtain GalNAc-LNP-siMKK4 nanoparticles with N/P molar ratios of 0, 2, 4, and 6. Among them, each nanoparticle contained 1 μg of siMKK4. Then, they were put into the gel electrophoresis device (Shanghai Qinxiang Scientific Instrument Co., Ltd, Chemi Scope 6200, China) for electrophoresis experiment. The electrophoresis condition was 100 V, 15 min, and the naked siMKK4 (1 μg) was used as the control.

The gene protective performance assessment: 2 μL RNase solution (12.5 μg/mL) was mixed with GalNAc-LNP-siMKK4 at N/P molar ratios of 0, 2, 4, and 6. After incubated at 37 °C for 3 h, 2 μL of 0.5 M EDTA solution was added and allowed to stand at room temperature for 10 min to terminate the enzymatic reaction. Then 5 μL of sodium dextran sulfate solution (10 mg/mL) was added and sonicated for 10 min in a 37 °C water bath for 2 h to displace siMKK4 from LNP. Then it was placed in a gel electrophoresis device (Shanghai Qinxiang Scientific Instrument Co., Ltd, Chemi Scope 6200, China) to detect the protective effect. The naked siMKK4 (1 μg) was used as the control.

### 2.8 Screen of GalNAc-PEG_2000_-DSPE ratio as targeting moiety of GalNAc

siNC-FAM was loaded into LNPs to detect the cellular uptake of LNPs. AML-12 cells were incubated with GalNAc-LNP-siNC-FAM at different molar proportions of GalNAc-PEG_2000_-DSPE (1, 5, 10%) and LNP-siNC-FAM without GalNAc-PEG_2000_-DSPE (0%) for 6 h. After trypsin digestion, the cells were collected and flow cytometry (Becton, Dickinson and Company, BD FACSAria, America) was used to detect the cellular uptake.

AML-12 cells were incubated with GalNAc-LNP-siMKK4 at different molar proportions of GalNAc-PEG_2000_-DSPE (1, 5, 10%) and LNP-siMKK4 without GalNAc-PEG_2000_-DSPE (0%) for 6 h. Then, fresh culture medium was replaced and qRT-PCR (Thermo Fisher Company, QuantStudio, Singapore) was used to detect the downregulation efficiency of siMKK4 after incubation for 24 h.

### 2.9 Preparation of LNP-siMKK4 and GalNAc-LNP-siMKK4

DOTAP, DSPC, Chol, and GalNAc-PEG_2000_-DSPE were dissolved in anhydrous ethanol at concentrations of 2 mg/mL, 2 mg/mL, 2 mg/mL, and 0.5 mg/mL, respectively. siMKK4 was dissolved in DEPC water at a concentration of 0.1 μg/μL. The siMKK4 solution was then added dropwise to the pre-mixed lipid solution, followed by sonication for 20 min and incubation at room temperature for 20 min. Ethanol was removed by rotary evaporation to obtain the LNP-siMKK4 or GalNAc-LNP-siMKK4 nanoparticles. The final lipid composition for LNP-siMKK4 was DOTAP:DSPC:Chol:GalNAc-PEG_2000_-DSPE = 50:10:40:0 (molar ratio). As for GalNAc-LNP-siMKK4, it was DOTAP:DSPC:Chol:GalNAc-PEG_2000_-DSPE = 50:5:40:5 (molar ratio). The final concentration of siMKK4 in both nanoparticle formulations was 0.09 μg/μL.

### 2.10 Characterization of GalNAc-LNP-siMKK4

Stability evaluation: GalNAc-LNP-siMKK4 and LNP-siMKK4 suspensions were added to 10% FBS and stored at 4 °C. The storage stability of LNP-siMKK4 and GalNAc-LNP-siMKK4 suspensions in 10% FBS was evaluated by nano laser particle size analyzer (Beckman Coulter, Inc, DelsaTM Nano C, America) at days 1, 3, 7, 10, 14 and 21, respectively.

Hemolysis assay: 0.8 mL of 2% red blood cell suspension was mixed evenly with 0.8 mL of purified water, saline, LNP-siMKK4, or GalNAc-LNP-siMKK4 (the final siMKK4 concentration was kept the same concentration as gene transfection). The mixture was allowed to stand at 37 °C for 3 h and then centrifuged (10 min, 3000 r/min, 4 °C) to collect the supernatant. The absorbance value of the supernatant was measured at a wavelength of 415 nm using a SpectraMax Absorbance Reader (Molecular Devices Corporation, 2100, America).

Cytotoxicity evaluation (live/dead cell assay): AML-12 cells cultured in a 12-well plate were treated with LNP-siMKK4 and GalNAc-LNP-siMKK4 (siMKK4 concentration of 50 nM) for 24 h. Then, 400 μL of AM/PI dye was added to each well and incubated for 20 min before observation under a fluorescence microscope (Nikon, Ts2R-FL, Japan).

Particle size, zeta potential, and morphological analysis: 0.8 mL of GalNAc-LNP-siMKK4 and LNP-siMKK4 suspensions were taken, and the average particle size, polydispersity index (PDI), and zeta potential were determined and recorded using a nano laser particle size analyzer (Beckman Coulter, Inc, DelsaTM Nano C, America) at room temperature. For morphological analysis, 10 μL of prepared GalNAc-LNP-siMKK4 was placed onto a copper grid. The grid was air-dried completely at room temperature to eliminate solvent residues and transferred into the sample chamber of transmission electron microscope (TEM) (Field Electron and Ion Company, FEI Tecnai G2 F20, America) to observe the morphology.

The encapsulation efficiency and gene loading capacity: The gray values of the gene bands in the agarose gel electrophoresis were quantified using ImageJ. The encapsulation efficiency and gene loading capacity were calculated using the following formula:

Encapsulationefficiency=(totalsiMKK4‐freesiMKK4)/(totalsiMKK4)×100%

Geneloadingcapacity=(weightofloadedsiMKK4)/(totalweightofsiMKK4‐loadedLNP)×100%

Release kinetics experiment: LNP-siNC-FAM and GalNAc-LNP-siNC-FAM nanoparticles were diluted with 2 mL of DEPC water, transferred to a dialysis bag (MWCO 20 kDa), and placed in 20 mL of pH 7.4 PBS solution. The mixture was then shaken at a constant temperature (37 °C, 120 r/min) on a shaker for 0.5, 1, 2, 4, 6, 8, 10, 12, 24, 48, 60, 72 and 96 h, respectively. 1 mL of dialysate was taken out and 1 mL of the same PBS solution was added. The fluorescence value was measured using a fluorescence spectrophotometer (Ex at 494 nm, Em at 522 nm). And the release amount of siNC-FAM was calculated based on the concentration and fluorescence intensity standard curve of siNC-FAM.

### 2.11 Competitive inhibition of cellular uptake

AML-12 cells (5 × 10^4^ cells/well) were added to 24-well cell plates with 12-mm cell slides, incubated at 37 °C for 12 h, and then replaced by the culture medium containing GalNAc (0 or 7 μM) at 37 °C for 1 h. After removal of the medium, the cells were treated with 1 mL of fresh medium containing LNP-siMKK4 or GalNAc-LNP-siMKK4. After incubation for 6 h at 37 °C, the cells were washed 3 times with PBS and fixed with 4% paraformaldehyde solution for 20 min. After the addition of DAPI (0.5 μg/mL) for 10 min, the cells were observed using confocal laser scanning microscopy (CLSM) (Olympus Corporation, FV3000, Japan).

### 2.12 Organ distribution and targeting ability *in vivo*

LNP-siNC-FAM or GalNAc-LNP-siNC-FAM (200 μL) was injected into the tail vein of mice. To observe the distribution in major organs, heart, liver, spleen, lung, and kidney were obtained at 1, 2, 6, and 12 h post-injection. All fluorescence images were obtained by small animal live imaging device (PerkinElmer, Inc, IVIS®Lumina S5, America) at excitation and emission wavelengths of 494 nm and 519 nm, respectively. For liver-specific analysis, liver tissue was collected 6 h post-injection and stored at −80 °C. Frozen liver sections were prepared and immunostained with HNF-4α antibody, CD68 antibody and CD31 antibody at 4 °C for 12 h, respectively. The sections were washed twice with 0.1% PBST (pH 7.4) and incubated with Alexa Fluor 594-labeled secondary antibody at room temperature for 2 h. After three washes with PBST, cell nucleus was stained with DAPI (1.0 μg/mL). Tissue sections were observed using CLSM (Olympus Corporation, FV3000, Japan), and image quantification was performed using embedded software.

### 2.13 Colony formation assay

AML-12 cells (2000 cells/well) were inoculated into a 6-well plate and cultured for 12 h. Subsequently, the cells were treated with CCl_4_ (80 μL 200 mM CCl_4_ were added to 1920 μL of DMEM/F12 culture medium to obtain 8 mM CCl_4_ solutions) for 4 h, while untreated cells were served as the control group. The original culture medium was removed from the wells, and cells were washed with PBS. Then, LNP-siNC, LNP-siMKK4, and GalNAc-LNP-siMKK4 (equivalent to 50 nM siMKK4) were prepared, diluted with DMEM/F12 medium to 1 mL, and added to the above 6-well plate. After 6 h of incubation, the medium was replaced with DMEM/F12 complete culture medium. Cells were washed and replaced with fresh DMEM/F12 medium every other day until visible colonies were formed. The cell culture medium was discarded, and cells were washed with PBS three times (3 min per wash). Cells were fixed with 4% paraformaldehyde (1 mL) at room temperature for 20 min. The paraformaldehyde solution was discarded, and 1% crystal violet staining solution was added and incubated for 20 min. Crystal violet was discarded, and cells were washed twice with PBS. After drying, colonies were photographed and recorded for formation analysis.

### 2.14 Ki67 immunofluorescence staining

AML-12 cells (5 × 10^4^ cells/well) were inoculated into a 24-well plate pre-loaded with 12-mm cell slides. After 12 h, the cells were treated with CCl_4_ (20 μL 200 mM CCl_4_ were added to 480 μL of DMEM/F12 culture medium to obtain 8 mM CCl_4_ solutions) for 4 h, while untreated cells were served as the control group. The CCl_4_-containing medium was replaced with fresh medium containing LNP-siNC, LNP-siMKK4, or GalNAc-LNP-siMKK4 (equivalent to 50 nM siMKK4) and incubated for 6 h. Subsequently, the medium was replaced with DMEM/F12 complete medium. After 48 h, cells were fixed with 4% paraformaldehyde for 20 min, permeabilized with 0.1% Triton X-100 for 10 min, and blocked with 5% serum at room temperature for 1 h. Cells were incubated overnight with Ki67 antibody at 4 °C. After three washes with PBST, Alexa Fluor 594-labeled secondary antibody was added and incubated at room temperature in the dark for 1 h. The cell nucleus was stained with 1 μg/mL DAPI for 5 min, and the samples were observed using CLSM (Olympus Corporation, FV3000, Japan).

### 2.15 MTT assay

LNP-siMKK4 and GalNAc-LNP-siMKK4 were diluted with DMEM/F12 medium to a concentration of 50 nM. AML-12 cells (5000 cells/well) were inoculated into a 96-well plate, followed by culture for 12 h. Then, cells were treated with CCl_4_ (8 μL 200 mM CCl_4_ were added to 192 μL of DMEM/F12 culture medium to obtain 8 mM CCl_4_ solutions) for 4 h, while untreated cells were served as the control group. The CCl_4_-containing medium was removed from the 96-well plate and respectively replaced with LNP-siNC, LNP-siMKK4, and GalNAc-LNP-siMKK4 at varying concentrations. After 6 h of incubation, the carrier solution was removed and replaced with DMEM/F12 complete medium. After 48 h of transfection, the nanoparticle solution in the 96-well plate was replaced with 5 mg/mL 20 μL MTT solution. After cells were incubated for 4 h, the solution was removed from each well, and 150 μL DMSO was added. Plates were shaken on a shaker for 10 min. The OD values were measured at 492 nm by SpectraMax Absorbance Reader (Molecular Devices Corporation, 2100, America), and the cell survival rate was calculated.

### 2.16 Immunofluorescence assay

AML-12 cells (5 × 10^4^ cells/well) were inoculated into a 24-well plate pre-loaded with 12-mm cell slides. After 12 h, the cells were treated with CCl_4_ (20 μL 200 mM CCl_4_ were added to 480 μL of DMEM/F12 culture medium to obtain 8 mM CCl_4_ solutions), while untreated cells were served as the control group. The CCl_4_-containing medium was replaced with fresh medium containing LNP-siNC, LNP-siMKK4, or GalNAc-LNP-siMKK4 (equivalent to 50 nM siMKK4) and incubated for 6 h. Subsequently, the medium was replaced with DMEM/F12 complete medium. After 48 h, cells were fixed with 4% paraformaldehyde for 20 min, permeabilized with 0.1% Triton X-100 for 10 min, and blocked with 5% serum at room temperature for 1 h. Primary antibodies against MKK4, p-MKK4, p-p38, p-MKK7, p-JNK1, p-ELK1, and p-ATF2 were incubated with cells overnight at 4 °C. After three washes with PBST, Alexa Fluor 594-labeled secondary antibodies were incubated to label primary antibodies against MKK4, p-MKK4, p-p38 and p-MKK7 at room temperature in the dark for 1 h. Alexa Fluor 488-labeled secondary antibodies were incubated to label primary antibodies against p-JNK1, p-ELK1, and p-ATF2 at room temperature in the dark for 1 h. Finally, the cell nucleus was stained with 1 μg/mL DAPI for 5 min, and the samples were observed using CLSM (Olympus Corporation, FV3000, Japan).

### 2.17 Western Blot experiment

AML-12 cells (1 × 10^5^ cells/well) were seeded into 6-well plates. After 12 h, the cells were treated with CCl_4_ (80 μL 200 mM CCl_4_ were added to 1920 μL of DMEM/F12 culture medium to obtain 8 mM CCl_4_ solutions) for 4 h, while untreated cells were served as the control group. The CCl_4_-containing medium was replaced with fresh medium containing LNP-siNC, LNP-siMKK4, or GalNAc-LNP-siMKK4 (equivalent to 50 nM siMKK4) and incubated for 6 h. Subsequently, the medium was replaced with DMEM/F12 complete medium and incubated for 48 h. The cells were harvested and lysed in ice-cold RIPA for 30 min. Proteins were collected by centrifugation at 12,000 r/min (4 °C, 15 min). Western Blot analysis was performed on the extracted proteins. The expression levels of MKK4, p-MKK4, p-p38, MKK7, p-MKK7, p-JNK1, p-ELK1, and p-ATF2 were detected and quantified using ImageJ software.

### 2.18 Therapeutic effect of GalNAc-LNP-siMKK4 on acute-on-chronic liver failure

Animal model of acute-on-chronic liver failure was conducted following the reported method [24,60]. Healthy mice were selected and randomly divided into 5 groups: Normal + PHx group (control group), CCl_4_ + PHx group (model group), CCl_4_ + PHx + LNP-siNC group (model + LNP-siNC group), CCl_4_ + PHx + LNP-siMKK4 group (model + LNP-siMKK4 group), and CCl_4_ + PHx + GalNAc-LNP-siMKK4 group (model + GalNAc-LNP-siMKK4 group). After adapting to the environment for one week, the control group was given physiological saline for four weeks, while the other groups were intraperitoneally injected with CCl_4_. 20% CCl_4_ oil solution (CCl_4_: soybean oil = 1:4) (0.5 mL/kg) was injected twice a week for a total of 4 weeks. On the first day of the fifth week, each group was given physiological saline, physiological saline, LNP-siNC, LNP-siMKK4, and GalNAc-LNP-siMKK4 *via* tail vein injection, with siNC or siMKK4 concentrations of 0.9 mg/kg. The left liver (30% of the liver volume) lobectomy was performed on the second day of the fifth week. On the third day of the fifth week, each group was given physiological saline, physiological saline, LNP-siNC, LNP-siMKK4, and GalNAc-LNP-siMKK4 *via* tail vein injection. At the end of treatment on the fifth day of the fifth week, the first batch of mice were euthanized to collect blood, heart, liver, spleen, lung, and kidney. The efficiency of liver regeneration in this batch was determined by liver photographs, hematoxylin & eosin (H&E) staining, Ki67 staining, and terminal deoxynucleotidyl transferase mediated deoxyuridine triphosphate nick-end labeling (TUNEL) staining. ImageJ software was also used to analyze the expression levels of MKK4, p-MKK4, p-p38, p-MKK7, p-JNK1, p-ELK1, and p-ATF2 in liver pathological sections to evaluate the therapeutic effects of different treatment groups. The collected blood was centrifuged at 3000 r/min (4 °C, 15 min) to obtain the supernatant serum. The blood biochemistry analyzer was detected the concentrations of alanine aminotransferase (ALT), aspartate transaminase (AST), albumin (ALB), blood ammonia (BA), urea, blood urea nitrogen (BUN), and creatinine (CR) in the serum. Finally, H&E staining of heart, spleen, lung, kidney was used to evaluate the potential impact *in vivo*. The second independent batch of mice underwent liver high-resolution 3D imaging using a small animal Micro-CT (PerkinElmer, Inc, Quantum GX, America) before and after PHx, as well as on the third and seventh day. Iodixanol (200 μL) contrast agent was injected into the tail vein to better depict the edges of the liver parenchyma. The liver volume was analyzed by using 3D volumetric software (Mimics 13.0, Materialise NV, Belgium).

### 2.19 Statistical analysis

The data were presented as mean ± standard deviation (SD). All statistical analyses were conducted using GraphPad Prism 9.0 (GraphPad Software, San Diego, CA, America). Comparisons between two groups were performed using the unpaired two-tailed Student's t-test. For comparisons among multiple groups, one-way analysis of variance (ANOVA) was applied, followed by Tukey's multiple-comparisons tests. Survival data were analyzed using Kaplan-Meier curves. Differences among all groups were assessed with the global log-rank test. Hazard ratios (HR) and 95% confidence intervals (CI) were derived from Cox proportional hazards models. Pre-specified pairwise comparisons were performed using the log-rank test with Bonferroni correction to account for multiple testing. A value of *p* < 0.05 was considered to show a statistically significant difference.

## 3 Results and discussion

### 3.1 Preparation and characterization of GalNAc-LNP-siMKK4

**Fig. 2:**
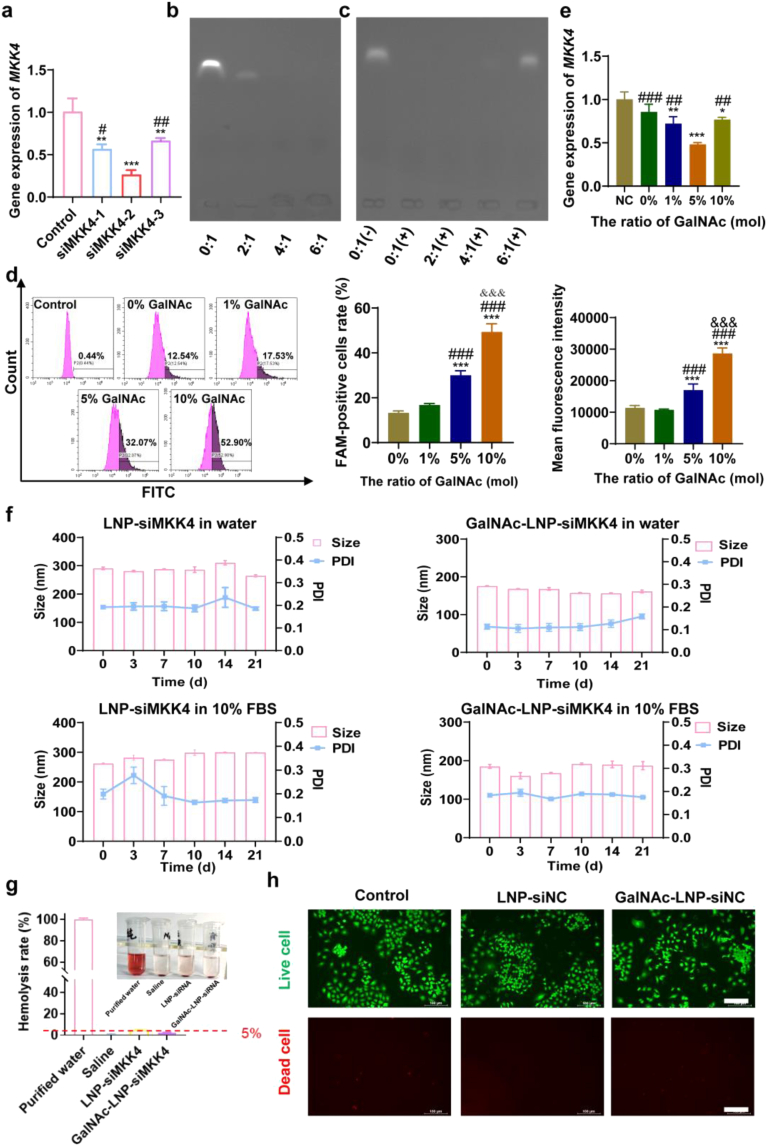
Optimization and characterization of GalNAc-LNP-siMKK4 nanoparticles. (a) Screening of effective sequences for siMKK4 (mean ± SD; *n* = 3), ∗∗ *p* < 0.01 and ∗∗∗ *p* < 0.001, compared to the control group, ^#^*p* < 0.05 and ^##^*p* < 0.01, compared to the siMKK4-2 group. Data were analyzed by one-way ANOVA with Tukey's multiple-comparisons tests. (b) The gene loading capacity of siMKK4 at different N/P ratio. (c) The gene protective performance of siMKK4 at different N/P ratio (+ represents the presence of RNase, - represents the absence of RNase). (d) Targeting efficiency of GalNAc-LNP-siMKK4 detected by flow cytometry and quantitative analysis of FAM-positive cells rate and mean fluorescence intensity. mean ± SD, *n* = 3, ∗∗∗*p* < 0.001, compared to 0% GalNAc, ^###^*p* < 0.001, compared to 1% GalNAc, ^&&&^*p* < 0.001, compared to 5% GalNAc. (e) Gene silence ability to *MKK4* in AML-12 cells by GalNAc-LNP-siMKK4 containing different molar proportions of GalNAc. mean ± SD, *n* = 3, ∗*p* < 0.05, ∗∗*p* < 0.01 and ∗∗∗*p* < 0.001, compared to NC group, ^##^*p* < 0.01 and ^###^*p* < 0.001, compared to 5% GalNAc. (f) Stability of LNP-siMKK4 and GalNAc-LNP-siMKK4 in water or 10% FBS. (g) Representative images of hemolysis assay and quantitative statistics of hemolysis rate. (h) Live/dead cell staining of AML-12 cells after different treatments (Scale bar = 100 μm).

qRT-PCR was used to screen the effective gene sequences of siMKK4 designed in Table S2. As shown in Fig. 2a, compared with siMKK4-1 and siMKK4-3, siMKK4-2 showed the best downregulation effect on *MKK4.* Therefore, the gene sequence siMKK4-2 was selected and shown as siMKK4 in the subsequent experiments. Gel electrophoresis assays were performed to evaluate the siMKK4-loading capacity of LNPs. As shown in Fig. 2b, when N/P ratio (n/n) was 4 or above, siMKK4 was fully loaded to form GalNAc-LNP-siMKK4. To further investigate the gene protective performance, after co-incubated with RNase and replaced siMKK4 with sodium sulfate dextran, gel electrophoresis assays were also performed (Fig. 2c). The results indicated that the N/P ratio (n/n) of 6 can effectively prevent siMKK4 from RNase degrading. In order to fully load the therapeutic gene and better resist nuclease degradation, the N/P ratio (n/n) of 6 was selected in the subsequent experiments.

To optimize the targeting efficiency, GalNAc-LNP-siMKK4 was prepared using different molar proportions of GalNAc-PEG_2000_-DSPE with targeting moiety of GalNAc (1%, 5%, 10%) and LNP-siMKK4 without GalNAc-PEG_2000_-DSPE (0%) by reverse phase evaporation method. The cellular uptake and the gene silence to *MKK4* in AML-12 cells were evaluated. The flow cytometry results (Fig. 2d) showed that GalNAc-LNP-siMKK4 containing targeting moiety of GalNAc was more uptake by AML-12 cells than without it. Adding GalNAc-PEG_2000_-DSPE will increase the targeting ability to AML-12 cells due to the high affinity of ASGPR and GalNAc. Further gene silence of *MKK4* in AML-12 cells using qRT-PCR (Fig. 2e) showed that GalNAc-LNP-siMKK4 prepared with a molar concentration of 5% GalNAc-PEG_2000_-DSPE exhibited the best silencing effect on *MKK4* compared to other groups. It is worth noting that although the formula containing 10% GalNAc-PEG_2000_-DSPE showed higher cellular uptake, the gene silencing effect was not as good as the formula containing 5% GalNAc-PEG_2000_-DSPE. This is because cellular uptake is only the first step in the gene transfection, and subsequent intracellular escape and gene release can also affect the final transfection effects. The high PEG density in 10% GalNAc-PEG_2000_-DSPE may be overly stable, resulting in high uptake but poor transfection. In contrast, the 5% formula seems to achieve better balance between cellular uptake and knockdown effect. Therefore, GalNAc-LNP-siMKK4 were prepared using 5% GalNAc-PEG_2000_-DSPE.

The nanoparticles were characterized by laser particle size analysis (Fig. S1). The results showed that LNP-siMKK4 had a particle size of 290.8 ± 4.6 nm and a zeta potential of 34.7 ± 0.73 mV, and GalNAc-LNP-siMKK4 exhibited a particle size of 175.9 ± 0.8 nm and a zeta potential of 20.4 ± 0.67 mV. TEM results indicated that GalNAc-LNP-siMKK4 had a uniform spherical structure (Fig. S2). The polydispersity index (PDI) (Fig. S3) of LNP-siMKK4 and GalNAc-LNP-siMKK4 indicated that LNP-siMKK4 and GalNAc-LNP-siMKK4 were uniformly dispersed (PDI <0.2). The storage stability of LNP-siMKK4 and GalNAc-LNP-siMKK4 in PBS and 10% FBS (Fig. 2f) indicated that LNP-siMKK4 and GalNAc-LNP-siMKK4 had good stability.

The results of the hemolysis assay (Fig. 2g) showed that both LNP-siMKK4 and GalNAc-LNP-siMKK4 induced less than 5% of hemolysis rate, indicating acceptable blood compatibility at the tested concentration. The results of MTT assay (Fig. S4) and live/dead cells staining (Fig. 2h) confirmed that the gene vectors exhibited low cytotoxicity to the cultured hepatocytes *in vitro*. The gene encapsulation efficiency of GalNAc-LNP-siMKK4 was 96.7%, and gene loading of GalNAc-LNP-siMKK4 was 2.59%. The release kinetics experiment found that the cumulative release of GalNAc-LNP-siNC-FAM nanoparticles was about 40% after 96 h, indicating a sustained release characteristic (Fig. S5).

### 3.2 In vitro and *in vivo* hepatocytes targeting ability of GalNAc-LNP-siMKK4

**Fig. 3:**
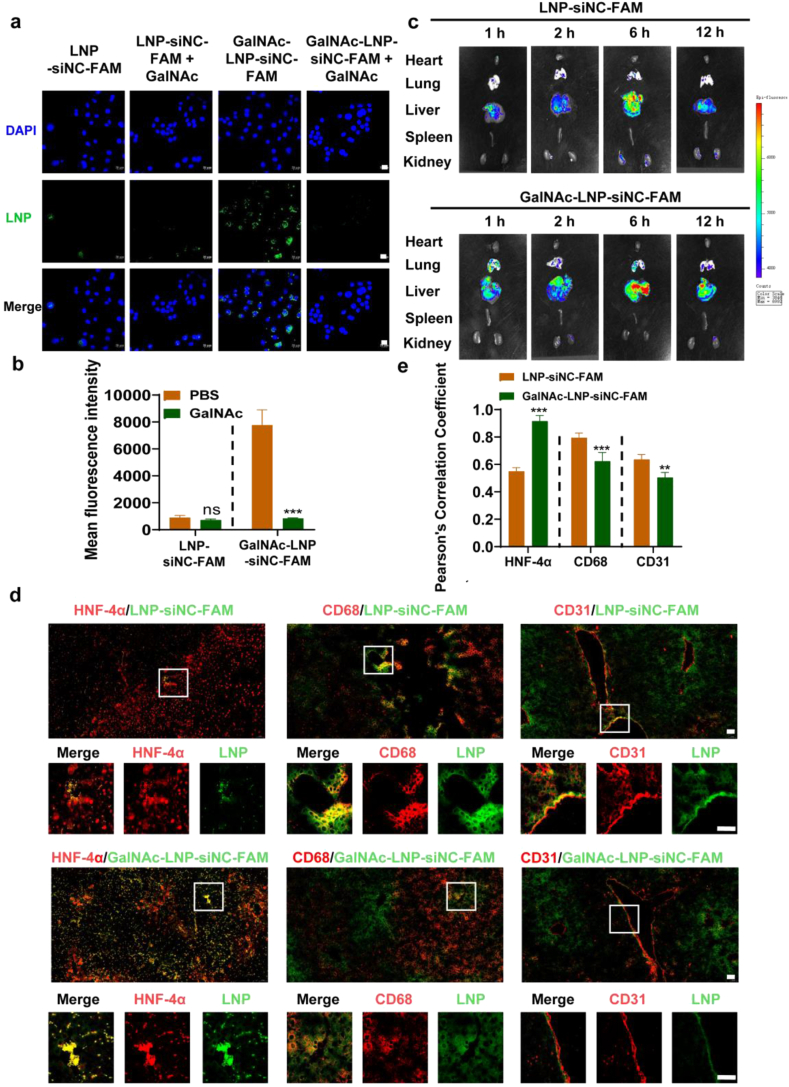
*In vitro* and *in vivo* liver tropism and hepatocytes targeting ability. (a) Cellular uptake of GalNAc-LNP-siNC-FAM by AML-12 cells with or without pre-incubation with GalNAc after 6 h (Scale bar = 50 μm). (b) Quantitative statistics of Fig. a. mean ± SD, *n* = 3, ns (no significant, *p* > 0.05), ∗∗∗*p* < 0.001, compared to the addition of GalNAc group. Data were analyzed by the unpaired two-tailed Student's t-test. (c) *In vitro* imaging of major organs. (d) Colocalization of HNF-4α/CD68/CD31 with LNP-siNC-FAM or GalNAc-LNP-siNC-FAM in the liver of mice (Scale bar = 50 μm). (e) Quantitative statistics of Fig. d. mean ± SD, *n* = 3, ∗∗*p* < 0.01 and ∗∗∗*p* < 0.001, compared to LNP-siNC-FAM group. Data were analyzed by the unpaired two-tailed Student's t-test.

Competitive inhibition of cellular uptake experiments were conducted using CLSM (Fig. 3a and b), and the results showed that in the absence of GalNAc, the fluorescence intensity of the GalNAc-LNP-siNC-FAM group was significantly stronger than that of the LNP-siNC-FAM group, indicating that GalNAc-modified GalNAc-LNP-siNC-FAM had better targeting properties for AML-12 cells. After treating with GalNAc, the fluorescence intensity of the GalNAc-LNP-siNC-FAM group significantly decreased, indicating that free GalNAc has a competitive inhibitory effect on the cellular uptake by AML-12 cells due to the recognition by GalNAc and ASGPR receptor.

To further determine whether it has liver targeting properties *in vivo*, FAM-labeled LNP-siNC-FAM or GalNAc-LNP-siNC-FAM was injected *via* tail vein to mice. The results were shown in Fig. 3c. Both LNP-siNC-FAM and GalNAc-LNP-siNC-FAM accumulated the most in the liver at 6 h, and GalNAc-LNP-siNC-FAM accumulated more in the liver than LNP-siNC-FAM, indicating that GalNAc-LNP-siNC-FAM has good liver tropism. At 12 h, GalNAc-LNP-siNC-FAM still accumulated a lot in the liver, indicating that GalNAc-LNP-siNC-FAM had a good retention effect in liver. In addition, HNF-4α, CD68, and CD31 fluorescence staining were respectively used as biomarker for hepatocytes, Kupffer cells and liver sinusoidal endothelial cells to further observe the cellular targeting ability to different liver cells. As shown in Fig. 3d and e, the fluorescence colocalization of HNF-4α and siNC-FAM overlapped more in the GalNAc-LNP-siNC-FAM group than in the LNP-siNC-FAM group. In contrast, the colocalization of siNC-FAM with CD68 and CD31 was more pronounced in the LNP-siNC-FAM group. The fluorescence intensity after treatment with nanoparticle without FAM fluorescent labeling was also tested to observe the effect of tissue autofluorescence. As shown in Fig. S6, the fluorescence intensity in mice without FAM labeling was almost invisible, and the colocalization with HNF-4α, CD68 and CD31 was also invisible. These results indicated that GalNAc modification can enhance cellular uptake by hepatocytes while reducing non-specific uptake by non-parenchymal liver cells. Therefore, GalNAc-LNP-siNC-FAM has better targeting ability to hepatocytes in the liver.

### 3.3 Role of GalNAc-LNP-siMKK4 in promoting cell proliferation *in vitro*

**Fig. 4:**
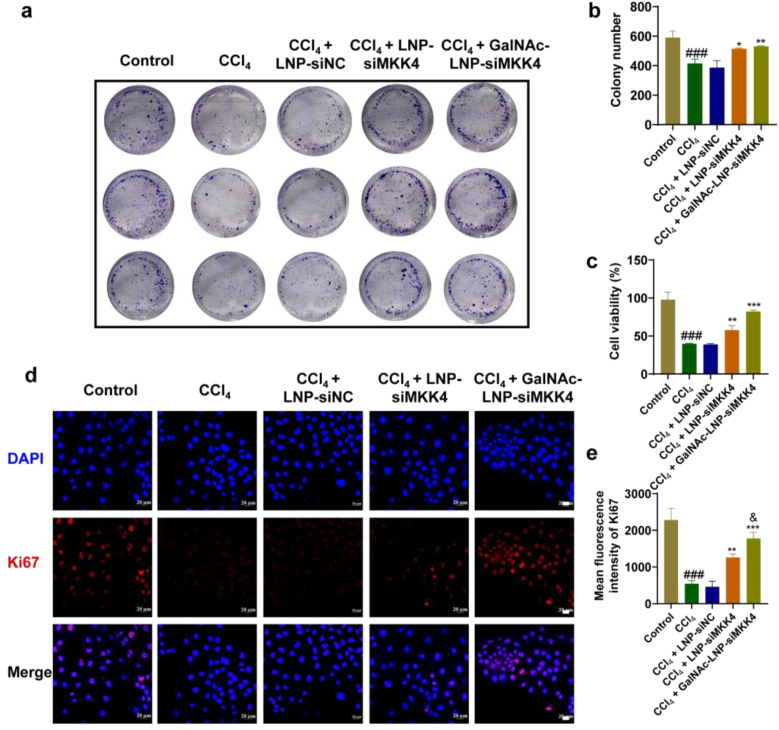
Proliferative activity on AML-12 cells after different treatments. (a) The proliferative effect evaluated by colony formation assay. (b) The quantitative statistics of Fig. a, mean ± SD, *n* = 3, ^###^*p* < 0.001, compared to control group, ∗*p* < 0.05 and ∗∗*p* < 0.01, compared to CCl_4_ group. Data were analyzed by one-way ANOVA with Tukey's multiple-comparisons tests. (c) The proliferative effect evaluated by MTT assay. mean ± SD, *n* = 3, ^###^*p* < 0.001, compared to control group, ∗∗*p* < 0.01 and ∗∗∗*p* < 0.001, compared to CCl_4_ group. Data were analyzed by one-way ANOVA with Tukey's multiple-comparisons tests. (d) Ki67 expression observed by CLSM (Scale bar = 20 μm). (e) The quantitative statistics of Fig. d. mean ± SD, *n* = 3, ^###^*p* < 0.001, compared to control group, ∗∗*p* < 0.01 and ∗∗∗*p* < 0.001, compared to CCl_4_ group, ^&^*p* < 0.05, compared to LNP-siMKK4 group. Data were analyzed by one-way ANOVA with Tukey's multiple-comparisons tests.

Colony formation assays were conducted to investigate the effects of GalNAc-LNP-siMKK4 on the proliferation ability of AML-12 cells. The assays (Fig. 4a and b) showed that pretreatment with CCl_4_ significantly reduced colony formation. Compared with the CCl_4_ group, there was no significant change in the area of cell clones in the LNP-siNC treatment group, indicating that LNP-siNC did not have a proliferative effect. However, after treatment with LNP-siMKK4 and GalNAc-LNP-siMKK4, the area of clone formation significantly increased, indicating that siMKK4-loaded LNP-siMKK4 and GalNAc-LNP-siMKK4 had good proliferative effect on AML-12 cells. Among them, GalNAc-LNP-siMKK4 had the largest colony area due to the targeting ability for enhancing intracellular siMKK4 delivery to exert the better proliferative effect. The MTT assay results (Fig. 4c) showed that the viability of AML-12 cells decreased to 39.84% after treatment with CCl_4_, while the cell viability increased to 82.20% after treatment with GalNAc-LNP-siMKK4, which was consistent with the cell cloning experiment.

CLSM was used to observe Ki67 and assess the proliferative effect of GalNAc-LNP-siMKK4 on AML-12 cells. As shown in Fig. 4d and e, compared with the other groups, the GalNAc-LNP-siMKK4 group had higher Ki67 expression, indicating that GalNAc-LNP-siMKK4 had a better proliferative effect.

### 3.4 Mechanism of GalNAc-LNP-siMKK4 in promoting cell proliferation *in vitro*

**Fig. 5:**
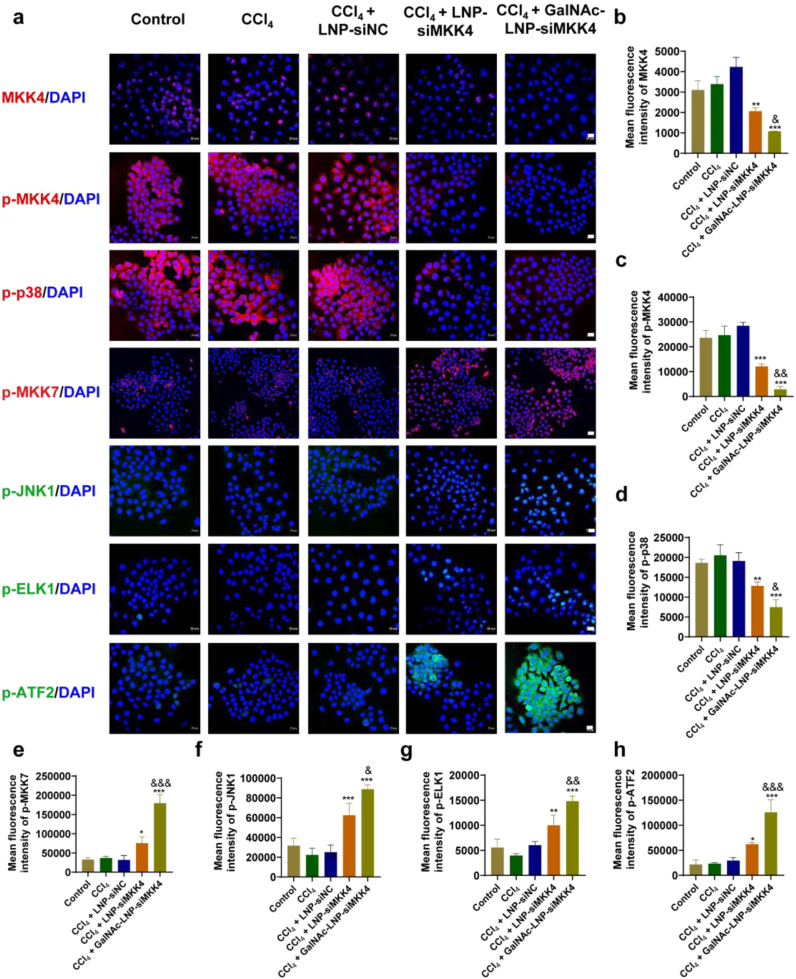
Immunofluorescence analysis of key signaling molecules in AML-12 cells after different treatments. (a) Immunofluorescence of mitogen-activated protein kinase kinase 4 (MKK4), phospho-mitogen-activated protein kinase kinase 4 (p-MKK4), phospho-p38 mitogen-activated protein kinase (p-p38), phospho-mitogen-activated protein kinase kinase 7 (p-MKK7), phospho-c-Jun N-terminal kinase 1 (p-JNK1), phospho-ETS transcription factor ELK1 (p-ELK1), and phospho-activating transcription factor 2 (p-ATF2) co-localized with DAPI (Scale bar = 20 μm). (b-h) Quantitative analysis of MKK4, p-MKK4, p-p38, p-MKK7, p-JNK1, p-ELK1, and p-ATF2 in AML-12 cells. mean ± SD, *n* = 3, ∗*p* < 0.05, ∗∗*p* < 0.01 and ∗∗∗*p* < 0.001, compared to CCl_4_ group, ^&^*p* < 0.05, ^&&^*p* < 0.01 and ^&&&^*p* < 0.001 compared to LNP-siMKK4 group. Data were analyzed by one-way ANOVA with Tukey's multiple-comparisons tests.

MKK4 is a protein kinase embedded in the main stress kinase signaling pathways p38, while MKK7 can activate the JNK pathway [24,[27], [28], [29]]. p38 and JNK signaling have antagonistic effects on hepatocyte proliferation and liver regeneration, with JNK signaling being considered proliferative and p38 signaling being anti-proliferative [24]. In response to environmental stress and carcinogenic signals, MKK7-JNK can promote cell proliferation, transformation, liver regeneration, and prevent premature aging. In contrast, activation of the MKK4-p38 pathway antagonizes the MKK7-JNK function. When MKK4 is inhibited, the signaling pathway from ASK1 to MKK4 is cut off, the apoptosis p38 signaling pathway is inhibited, and ASK1 cannot transmit signals to MKK4 normally [25,26]. But ASK1 can also activate MKK7 to continue transmitting signals downstream, and the ASK1-MKK7-JNK pathway can continue to function, so inhibiting MKK4 will promote liver regeneration. Immunofluorescence analyses validated the proliferative mechanism of GalNAc-LNP-siMKK4 (Fig. 5 and Fig. S7). After treatment with GalNAc-LNP-siMKK4, the expression of MKK4 was downregulated (Fig. 5b and Fig. S7), resulting in less activation of MKK4 in the form of phosphorylation (p-MKK4) (Fig. 5c and Fig. S7). The decrease of p-MKK4 further led to the reduced level of phosphorylated p38 (p-p38) (Fig. 5d and Fig. S7). p38 is a kinase associated with apoptosis, and the decline of p-p38 was correlated with an attenuation of downstream apoptotic signals. At the same time, more MKK7 was activated to p-MKK7 (Fig. 5e and Fig. S7), causing JNK1 (Fig. 5f and Fig. S7), ELK1 (Fig. 5g and Fig. S7) and ATF2 (Fig. 5h and Fig. S7) to be activated, leading to an increase in proliferation signals.

**Fig. 6:**
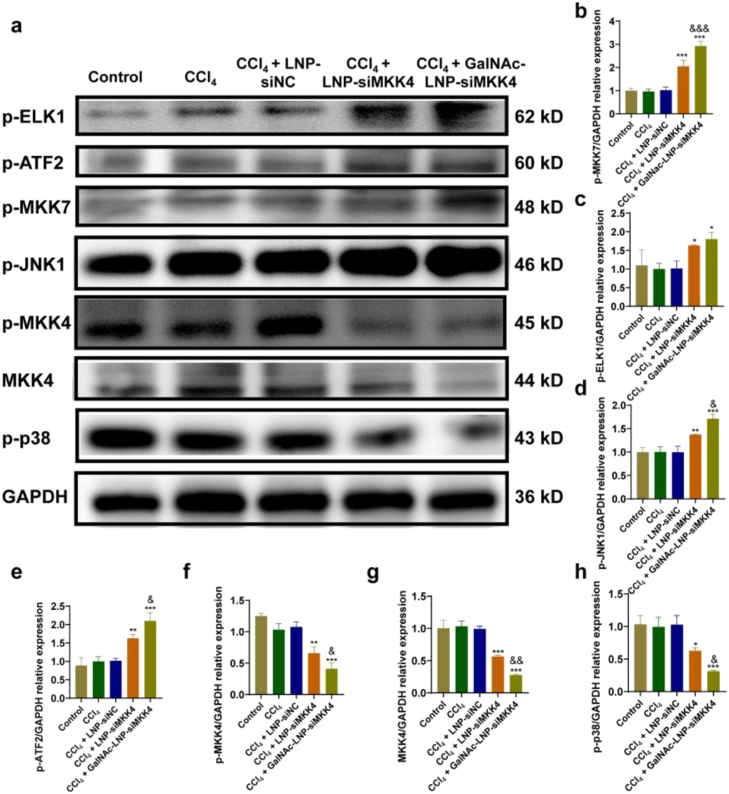
Western Blot analysis of p-ELK1, p-ATF2, p-MKK7, p-JNK1, p-MKK4, MKK4 and p-p38 in AML-12 cells after different treatments. (a) Western Blot analysis in AML-12 cells. (b-h) Quantitative analysis on the protein expression of p-ELK1, p-ATF2, p-MKK7, p-JNK1, p-MKK4, MKK4 and p-p38. mean ± SD, *n* = 3, ∗*p* < 0.05, ∗∗*p* < 0.01 and ∗∗∗*p* < 0.001, compared to CCl_4_ group, ^&^*p* < 0.05 and ^&&^*p* < 0.01, compared to LNP-siMKK4 group. Data were analyzed by one-way ANOVA with Tukey's multiple-comparisons tests.

The results of the Western Blot experiment are consistent with the immunofluorescence assay (Fig. 6). In brief, when MKK4 is down regulated by siMKK4, the MKK4-p38 apoptotic pathway is inhibited and the MKK7-JNK pathway is upregulated, thus disrupting the balance to induce the cells in a proliferative state. In addition, the total protein level of MKK7 remained unchanged (Fig. S8), indicating that GalNAc-LNP-siMKK4 can selectively downregulated MKK4 without causing off-target inhibition of MKK7.

### 3.5 Therapeutic effect of GalNAc-LNP-siMKK4 on acute-on-chronic liver failure of mice

**Fig. 7:**
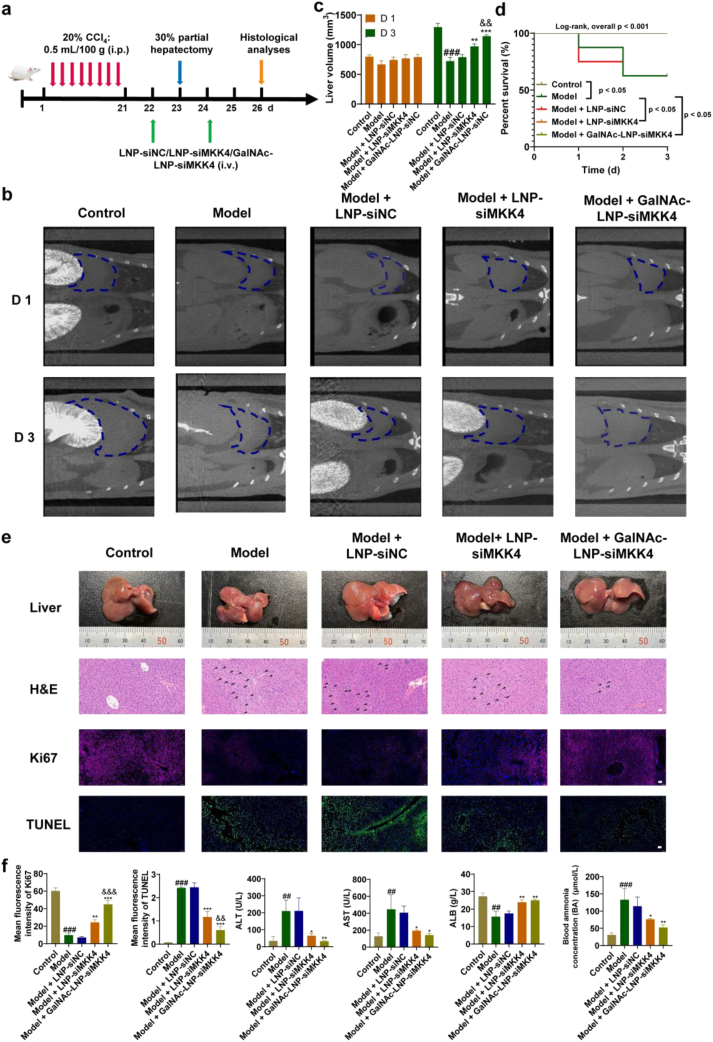
Therapeutic effect on acute-on-chronic liver failure of mice after different treatments. (a) Flowchart for the model establishment and drug treatment. Red arrow: intraperitoneal injection of CCl_4_, blue arrow: hepatectomy, green arrow: injection of nanoparticles, orange arrow: histological and biochemical analyses. (b) Representative screenshot of liver imaging using a small animal Micro-CT. (c) Liver volume calculated based on quantitative segmentation of CT data. mean ± SD, *n* = 6, ^###^*p* < 0.001, compared to control group, ∗∗*p* < 0.01 and ∗∗∗*p* < 0.001, compared to model group, ^&&^*p* < 0.01, compared to LNP-siMKK4 group. Data were analyzed by one-way ANOVA with Tukey's multiple-comparisons tests. (d) Kaplan-Meier survival curves of mice analyzed using the global log-rank test (*n* = 8 per group, *p* < 0.001). Differences among different treatments and details of hazard ratios (HR), 95% confidence intervals (CI) from pre-specified pairwise comparisons were provided in Supplementary Table S3. (e) Images of liver tissue and H&E (The black arrow indicates damaged hepatocytes.), Ki67, and TUNEL immunostaining (Scale bar = 50 μm). (f) Quantitative analysis of Ki67, TUNEL images and the concentrations of ALT, AST, ALB and BA in the serum. mean ± SD, *n* = 6, ^##^*p* < 0.01 and ^###^*p* < 0.001, compared to control group, ∗*p* < 0.05, ∗∗*p* < 0.01 and ∗∗∗*p* < 0.001, compared to model group, ^&&^*p* < 0.01 and ^&&&^*p* < 0.001, compared to LNP-siMKK4 group. Data were analyzed by one-way ANOVA with Tukey's multiple-comparisons tests.

Animal experiment was performed following the reported method [24,60]. After intraperitoneal injection of CCl_4_ for four weeks, the liver of mice was conducted to Masson's trichrome staining (Fig. S9). The results showed extensive deposition of blue collagen fibers in the CCl_4_ treatment group. The collagen area percentage was quantitatively evaluated, and showed a significant increase compared to the control group, indicating the chronic injury in the liver after CCl_4_ treatment. After the liver resection in chronic liver injury mice, ACLF model was established and different treatments were performed (Fig. 7a). High-resolution 3D imaging using a small animal Micro-CT was used to evaluate the liver volume. The results were shown in Fig. 7b and c. On the first day, there was no significant difference in liver volume among the groups. However, on the third day, the liver volume of mice in the LNP-siMKK4 group and GalNAc-LNP-siMKK4 group was significantly larger than that of the model group, and the liver volume in the GalNAc-LNP-siMKK4 group was larger than that of in the LNP-siMKK4 group. It is worth noting that in the GalNAc-LNP-siMKK4 treatment group, the liver volume of mice continued to increase on the 7th day compared to the 3rd day (Fig. S10), indicating that liver regeneration under drug treatment was sustained after early proliferation. The survival curves of mice after different treatments was evaluated by Kaplan-Meier analysis. The overall survival distributions across the five groups were significantly different (Fig. 7d). The survival rates of mice in the model group and LNP-siNC group were both 62.5%, while the survival rate of the mice treated with GalNAc-LNP-siMKK4 was 100%. Critically, the GalNAc-LNP-siMKK4 group exhibited a substantially lower risk of death compared to the model group (GalNAc-LNP-siMKK4 vs. model: Hazard Ratio (HR) = 0.027, 95% Confidence Interval (CI) = 0.002 - 0.343, *p* < 0.05) (Table S3). In addition, the liver surfaces of the model and LNP-siNC groups became rough and dull, and the liver surfaces turned white and uneven (Fig. 7e). The GalNAc-LNP-siMKK4 group had a rosy and glossy liver, and an increased of liver to body weight rate and liver regeneration rate compared to the model group (Fig. S11), indicating a significant promoting effect on liver regeneration. H&E staining confirmed that GalNAc-LNP-siMKK4 could protect the liver microstructure and significantly reduce the necrotic area of hepatocytes (Fig. 7e). Ki67 positive cell signals showed that the number of proliferating cells in GalNAc-LNP-siMKK4 group was 5 times that of model group and 2 times that of LNP-siMKK4 group (Fig. 7e and f). Meanwhile, TUNEL staining revealed that GalNAc-LNP-siMKK4 treatment effectively reduced the number of apoptotic cells to 1/4 of the model group, only half of the LNP-siMKK4 group (Fig. 7e and f). These results indicated that GalNAc-LNP-siMKK4 was more effective than LNP-siMKK4 in reducing cell apoptosis and promoting liver regeneration for treatment of acute-on-chronic liver failure of mice.

ALT and AST are the most commonly used indicators reflecting hepatocellular damage in clinical, with elevated serum levels indicating potential hepatocyte damage. The results in Fig. 7f showed that AST and ALT significantly increased in the mice of model group, while treatment with GalNAc-LNP-siMKK4 effectively restored liver function. ALB can reflect the synthesis reserve function of liver, which is a main function of the liver. The results in Fig. 7f indicated that liver synthesis reserve function was restored after treatment with GalNAc-LNP-siMKK4. The main cause of ACLF is the destruction of hepatocytes function, which leads to a decrease in urea synthesis and ineffective clearance of BA, resulting in an increase in BA levels. Therefore, BA levels are one of the indicators for evaluating the occurrence of ACLF. The results in Fig. 7f indicated that ACLF occurred in the mice of model group, resulting in abnormal BA levels. After treatment with GalNAc-LNP-siMKK4, BA levels returned to the normal range.

### 3.6 Mechanism of GalNAc-LNP-siMKK4 in the treatment of ACLF

**Fig. 8:**
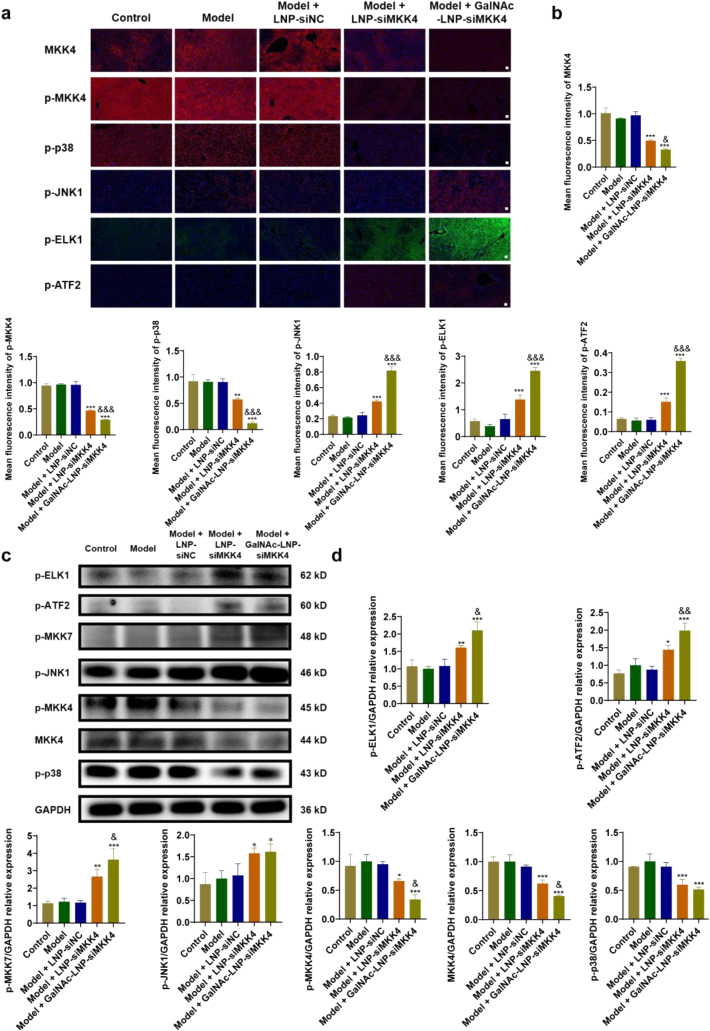
Immunofluorescence and Western Blot analysis of proliferative signaling on acute-on-chronic liver failure of mice after different treatments. (a) CLSM of MKK4, p-MKK4, p-p38, p-JNK1, p-ELK1, and p-ATF2 in liver tissue (Scale bar = 50 μm). (b) Quantitative analysis of MKK4, p-MKK4, p-p38, p-JNK1, p-ELK1 and p-ATF2 in liver tissue, mean ± SD, *n* = 6, ∗∗*p* < 0.01 and ∗∗∗*p* < 0.001, compared to model group, ^&^*p* < 0.05 and ^&&&^*p* < 0.001, compared to LNP-siMKK4 group. Data were analyzed by one-way ANOVA with Tukey's multiple-comparisons tests. (c) Western Blot analysis of the expression of p-ELK1, p-ATF2, p-MKK7, p-JNK1, p-MKK4, MKK4 and p-p38 in liver tissues. (d) Quantitative analysis of the protein expression in liver tissue. mean ± SD, *n* = 6, ∗*p* < 0.05, ∗∗*p* < 0.01 and ∗∗∗*p* < 0.001, compared to model group, ^&^*p* < 0.05 and ^&&^*p* < 0.01, compared to LNP-siMKK4 group. Data were analyzed by one-way ANOVA with Tukey's multiple-comparisons tests.

To explore in depth and intuitively the mechanism of GalNAc-LNP-siMKK4 in promoting liver regeneration, immunofluorescence staining and Western Blot assay were performed (Fig. 8). In normal liver, the MKK4-p38 (apoptotic) and the MKK7-JNK (proliferative) pathway are in a dynamic equilibrium state [20,24]. If the MKK4-p38 pathway is inhibited, the balance will be disrupted, and the MKK7-JNK pathway will play a dominant role, initiating liver regeneration. After treatment with GalNAc-LNP-siMKK4, the expression of MKK4 was downregulated (Fig. 8b and d), resulting in less activation of MKK4 in the form of phosphorylation, which means that p-MKK4 was decreased. As an upstream kinase of p38, p-MKK4 decreased, leading to less activation of p38 phosphorylation. p38 plays a key role in the apoptotic signaling pathway. A decrease in the level of phosphorylated form was associated with an attenuation of apoptotic signaling. At the same time, more MKK7 was activated in phosphorylated form, causing JNK1 and downstream transcription factors ELK1 and ATF2 to be activated, leading to an increase in proliferation signals. Notably, GalNAc-LNP-siMKK4 did not affect the total protein expression of MKK7, indicating that GalNAc-LNP-siMKK4 did not cause off-target effect on MKK7 (Fig. S12). To further investigate the relationship between the changes in the MKK4-p38 and MKK7-JNK pathways and liver regeneration, SP600125 (JNK1 inhibitor) and dehydrocorydaline chloride (p38 agonist) was used respectively. As shown in Fig. S13, the cellular viability of AML-12 cells decreased in CCl_4_ group. After treatment with GalNAc-LNP-siMKK4, the cellular viability was significantly enhanced, indicating a proliferative activity of GalNAc-LNP-siMKK4. However, after treatment with GalNAc-LNP-siMKK4 and JNK1 inhibitor/p38 agonist, the proliferative effect of GalNAc-LNP-siMKK4 was significantly reduced. The results indicated that the proliferative effect of GalNAc-LNP-siMKK4 was dependent on the activation of JNK1 and the inhibition of p38. Therefore, the therapeutic effect of GalNAc-LNP-siMKK4 is closely associated with the MKK4-p38 and the MKK7-JNK signaling pathways as the inhibition of MKK4 reported previously [[23], [24], [25], [26], [27], [28], [29]].

To preliminarily assess the potential impact of GalNAc-LNP-siMKK4 on major organs during the treatment period, histopathological examination, immunohistochemical analysis of main organs and biochemical test were performed. H&E staining of the main organs showed that the microstructure of the tissue was normal and there was no pathological damage in heart, spleen, lung, and kidney (Fig. S14a). Given the certain distribution of nanoparticles in the lung (Fig. 3c), potential pulmonary immune response was further evaluated by immunohistochemical staining. The results showed no significant increase in CD163-positive macrophage in the lung tissue after GalNAc-LNP-siMKK4 administration compared to the control group during the treatment period (Fig. S14b). In addition, blood biochemical tests were evaluated, as shown in Fig. S14c–e. Compared to the control group, there were no significant differences in the levels of UREA, BUN, and CR after treatment with LNP-siNC, LNP-siMKK4, and GalNAc-LNP-siMKK4, indicating that each nanoparticle did not cause significant damage to kidney function during the treatment period.

## 4 Discussion

A dual-targeted gene therapy system GalNAc-LNP-siMKK4 has developed as liver regeneration promoting strategy for the treatment of acute-on-chronic liver failure, which prepared by reverse phase evaporation method. This system is capable of treating acute-on-chronic liver failure in mice by promoting liver regeneration. In terms of targeting efficiency, conventional LNPs can accumulate in the liver primarily through passive targeting. However, they lack the ability to distinguish different hepatic cell types in liver, leading to non-specific cellular uptake by liver cells and consequently limited targeting specificity toward hepatocytes [61]. In contrast, GalNAc-siRNA conjugates achieve active targeting of hepatocytes by specifically binding to the ASGPR highly expressed on the surface of hepatocyte, thus offering higher and specific cellular uptake by hepatocyte [59]. The GalNAc-LNP-siMKK4 system developed in this study leverages the inherent liver tropism of LNPs and further incorporates the hepatocyte-specific targeting ligand GalNAc on LNPs, achieving dual targeting at both the organ and cellular levels for more precise hepatocyte delivery. This dual targeting design was confirmed by biodistribution studies *in vivo*, which demonstrated that GalNAc-LNP-siMKK4 exhibited greater and more prolonged hepatic accumulation compared to non-targeted LNP counterpart (Fig. 3). GalNAc-LNP-siMKK4 can achieve efficient gene delivery to liver parenchymal cells by combining the liver tropism of LNPs and modifying GalNAc targeting groups on LNPs through a dual targeting mechanism.

Apart from targeting capability, the stability of the delivery system is another critical determinant for therapeutic efficacy. Conventional LNPs can protect siRNA from degradation by nucleases through physical encapsulation within the core, thereby forming a protective barrier [59]. The GalNAc-siRNA conjugates can enhance the resistance of siRNA to ribonuclease degradation through chemical modification, providing a certain stability in blood circulation [59,62]. In this study, the constructed GalNAc-LNP-siMKK4 protects siRNA from enzymatic degradation through the physical encapsulation provided by the LNP. This system has been demonstrated to possess substantial resistance to RNase degradation (Fig. 2c) and to maintain good stability in the presence of serum (Fig. 2f).

Conventional LNPs, due to their inability to precisely distinguish between hepatic cell types, can cause off-target cytotoxicity [63]. In contrast, for GalNAc-siRNA conjugates, the exogenous siRNA is directly exposed in the bloodstream and may be recognized by pattern recognition receptors (PRRs), potentially triggering an immune response [64]. Compared to these two delivery systems, the GalNAc-LNP-siMKK4 system offers potential advantages: the encapsulation of siRNA within the LNP core may reduce the immunogenic risk associated with exposed nucleic acids, while the active targeting mediated by the GalNAc ligand is designed to minimize the non-specific distribution and associated cytotoxicity to other liver cells. During the treatment period, GalNAc-LNP-siMKK4 caused no significant histopathological damage to major organs (Fig. S14). The protein expression of homologous MKK7 was also evaluated by Western Blot analysis. As shown in Fig. S8 and Fig. S12, GalNAc-LNP-siMKK4 did not significantly affect MKK7 protein levels either *in vitro* or *in vivo*, providing initial evidence for the sequence-specificity of the siRNA. For future study of GalNAc-LNP-siMKK4, a systematic safety evaluation, including transcriptome-wide off-target analysis, full immunotoxicity profiling (complement activation, cytokine release, coagulation function), and long-term toxicology studies will be essential.

In terms of promoting liver regeneration, the MTT assay showed that the viability of AML-12 cells decreased to 39.84% after treatment with CCl_4_, while the cell viability increased to 82.20% after treatment with GalNAc-LNP-siMKK4. In addition, colony formation assay and Ki67 assay also demonstrated that GalNAc-LNP-siMKK4 had a good effect on promoting cell proliferation. After the ACLF mice model was established and performed with different treatments, CT was used to evaluate the liver volume. The liver volume of mice in the GalNAc-LNP-siMKK4 group was significantly larger than that of the model group. The survival rates of mice in the model group were 62.5%, while the survival rate of the mice treated with GalNAc-LNP-siMKK4 was 100%. Moreover, H&E staining, ALT, AST, ALB, and BA also demonstrated that GalNAc-LNP-siMKK4 had good gene therapy efficacy on promoting liver regeneration *in vivo*, reducing hepatocytes apoptosis, and promoting liver function recovery. Western Blot assay and immunofluorescence assay were further used to investigate the mechanism of liver regeneration. Both *in vivo* and *in vitro* experiments had shown that GalNAc-LNP-siMKK4 can downregulate the MKK4-p38 pathway (apoptotic pathway), and upregulate the MKK7-JNK pathway (cell proliferation). ASK1 is a kinase of both MKK4 and MKK7. Inhibiting the expression of MKK4 will lead to compensatory activation of MKK7 by ASK1 through phosphorylation. The activated MKK7 subsequently activates JNK1, which then further activates the downstream transcription factors ATF2 and ELK1, initiating the gene expression of cell proliferation. Meanwhile, reduced expression of MKK4 can attenuate the activation of p38, thereby diminishing p38-mediated pro-apoptosis and growth-suppress. This combined attenuation of apoptotic signals and enhancement of pro-proliferative signals promote hepatocyte proliferation, ultimately leading to liver regeneration, liver function recovery, and reduced mortality rate in mice. In current study, the critical initiation and proliferation phase (early postoperative) were focused, and the full therapeutic effect on liver injury during longer treatment period (7-14 days) will be essential in the clinical translation.

## 5 Conclusion

In summary, a dual targeted gene therapy system GalNAc-LNP-siMKK4 was developed to enhance liver regeneration for the treatment of acute-on-chronic liver failure. Based on the role of MKK4 in promoting the proliferation of hepatocytes, and the advantages of gene therapy of siRNA, siMKK4 was used for the first time in promoting liver regeneration. Specific inhibition of MKK4 expression can avoid the off-target effect of small molecular MKK4 inhibitor. Guided by the liver tropism of LNPs and targeting group of GalNAc, GalNAc-LNP-siMKK4 can efficiently bind to ASGPR, which is highly expressed on the surface of hepatocytes. By optimizing the ratio of targeting group GalNAc, GalNAc-LNP-siMKK4 could effectively address ACLF based on reducing expression of MKK4 to promote hepatocytes proliferation specifically mediated by the targeting moiety of GalNAc. GalNAc-LNP-siMKK4 has targeting ability to deliver therapeutic genes to hepatocytes, achieving a highly efficient gene therapy for promotion of liver regeneration and providing new therapeutic strategies in the clinical practice.

## Ethics approval and consent to participate

All experimental studies and animal care were performed in accordance with the guidelines and endorsement of the Animal Ethics Committee of the Fourth Military Medical University (NO: 20,250,099).

## CRediT authorship contribution statement

**Xiao-Pei Zhai:** Data curation, Formal analysis, Investigation, Methodology, Resources, Software, Validation, Visualization, Writing – original draft. **Jie-Hua Xing:** Data curation, Investigation, Methodology, Resources, Visualization, Writing – original draft. **Li-Shuang Hou:** Data curation, Investigation, Methodology, Resources, Validation, Visualization. **Tang-Rui Zhang:** Investigation, Methodology, Software, Visualization. **Wei He:** Conceptualization, Data curation, Methodology, Software, Supervision, Writing – review & editing. **Li-She Gan:** Conceptualization, Data curation, Investigation, Methodology, Software, Visualization. **Si-Yuan Zhou:** Data curation, Methodology, Software, Visualization. **Bang-Le Zhang:** Conceptualization, Data curation, Funding acquisition, Methodology, Project administration, Resources, Supervision, Writing – review & editing.

## Declaration of competing interest

The authors declare that they have no known competing financial interests or personal relationships that could have appeared to influence the work reported in this paper.

## Data Availability

Data will be made available on request.

## Abbreviations:

ACLF: acute-on-chronic liver failure
ALB: albumin
AML-12: alpha mouse liver 12
ASGPR: asialoglycoprotein receptor
ASK1: apoptosis signal-regulating kinase 1
AST: aspartate transaminase
BA: blood ammonia
BUN: blood urea nitrogen
CR: creatinine;
CT: computed tomography
EDTA: ethylene diamine tetraacetic acid
FBS: fetal bovine serum
GalNAc: N-acetylgalactosamine;
H&E: hematoxylin & eosin
HRP: horseradish peroxidase
IgG: immunoglobulin G
LNPs: lipid nanoparticles
MAP2: mitogen-activated protein 2
MAPK: mitogen-activated protein kinase
MKK4: mitogen-activated protein kinase 4
MKK7: mitogen-activated protein kinase 7
MTT: methylthiazolyldiphenyl-tetrazolium bromide;
PDI: polydispersity index
PHx: partial hepatectomy
p-ATF2: phospho-activating transcription factor 2
p-ELK1: phospho-ETS transcription factor ELK1
p-JNK1: phospho-c-Jun N-terminal kinase 1
p-MKK4: phospho-mitogen-activated protein kinase 4
p-MKK7: phospho-mitogen-activated protein kinase 7
p-p38: phospho-p38 mitogen-activated protein kinase
qRT-PCR: quantitative reverse-transcription polymerase chain reaction
RIPA: radioimmunoprecipitation assay
RNase: ribonuclease
SAPK: stress-activated protein kinas
siRNA: small interfering RNA
TEM: transmission electron microscopy
TUNEL: terminal deoxynucleotidyl transferase mediated dutp nick-end labeling
