# Supplementary material for: Dual targeted gene delivery strategy mediated by GalNAc-modified lipid nanoparticles enhances liver regeneration through specific knockdown of MKK4
Source: Mater Today Bio. 2026 Mar 24;38:103059. doi: 10.1016/j.mtbio.2026.103059 (PMC13067123; doi:10.1016/j.mtbio.2026.103059)
Supplement: Multimedia component 1 [file mmc1.pdf]

Supporting Information

Dual targeted gene delivery strategy mediated by GalNAc-modified lipid nanoparticles enhances liver regeneration through specific knockdown of MKK4

Xiao-Pei Zhai[1], Jie-Hua Xing[1], Li-Shuang Hou[1], Tang-Rui Zhang[1], Wei He[2,3], Li-She Gan[4*], Si-Yuan Zhou[1,2], Bang-Le Zhang[1,2 *]*

[1]Department of Pharmaceutics, School of Pharmacy, Fourth Military Medical University, Xi'an, 710032, China; [2]Key Laboratory of Gastrointestinal Pharmacology of the State Administration of Traditional Chinese Medicine, Fourth Military Medical University, Xi'an, 10032, China; [3]Department of Chemistry, School of Pharmacy, Fourth Military Medical University, Xi'an, 710032, China; [4]School of Pharmaceutical Science, Zhejiang Chinese Medical University, Hangzhou 311402, China.

***Correspondence to:** blezhang@fmmu.edu.cn (BL Zhang); weihechem@fmmu.edu.cn (W He); lsgan@zcmu.edu.cn (LS Gan).

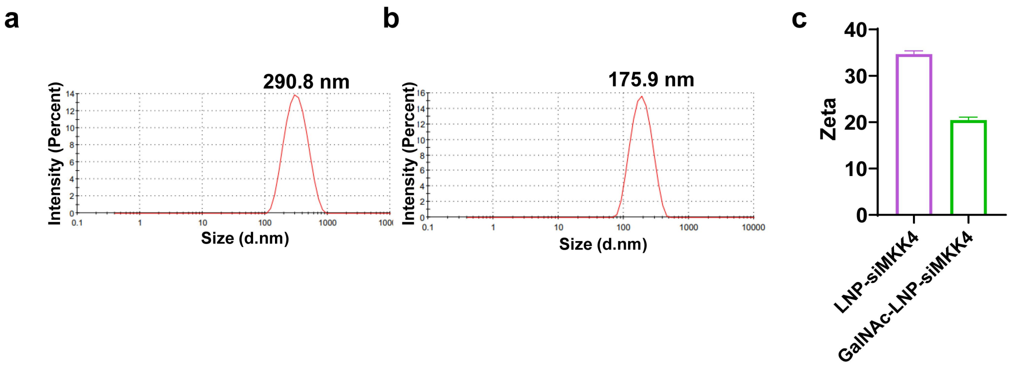

Fig. S1. Characterization of GalNAc-LNP-siMKK4 and LNP-siMKK4 nanoparticles. (a) Particle size distribution of LNP-siMKK4. (b) Particle size distribution of GalNAc-LNP-siMKK4. (c) Zeta potential of LNP-siMKK4 and GalNAc-LNP-siMKK4 (n = 3).

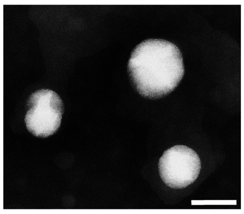

Fig. S2. TEM of GalNAc-LNP-siMKK4 (Scale bar = 100 nm)

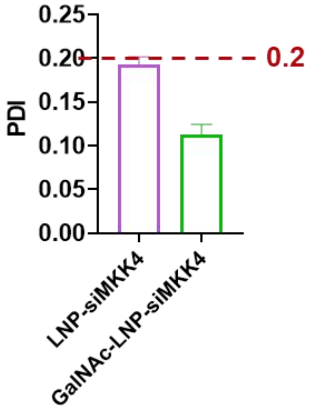

Fig. S3. Polydispersity index (PDI) of LNP-siMKK4 and GalNAc-LNP-siMKK4 (n = 3).

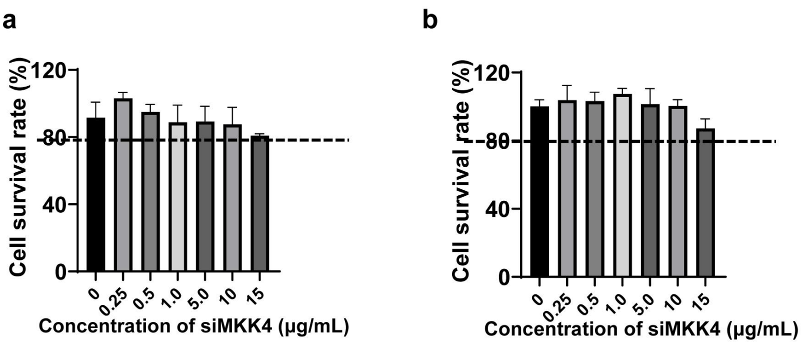

Fig. S4. Cytotoxicity evaluation of LNP-siNC and GalNAc-LNP-siNC by MTT (*n* = 3)

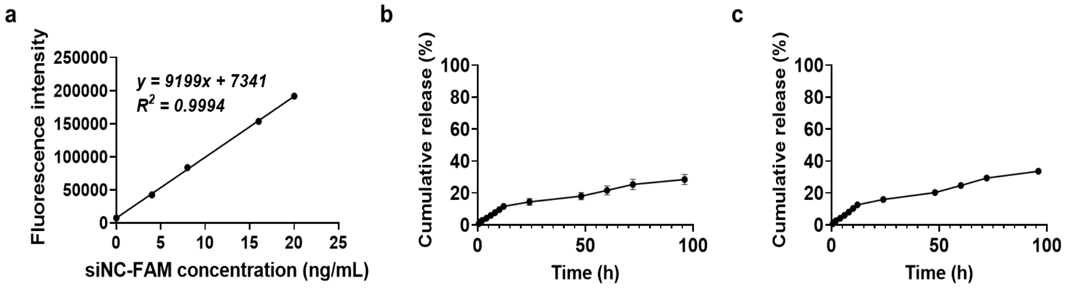

Fig. S5. The cumulative release curve of LNP-siNC-FAM and GalNAc-LNP-siNC-FAM. (a) Standard curve of siNC-FAM. (b) Cumulative release of LNP-siNC-FAM in PBS. *n* = 3. (c) Cumulative release of GalNAc-LNP-siNC-FAM in PBS. *n* = 3.

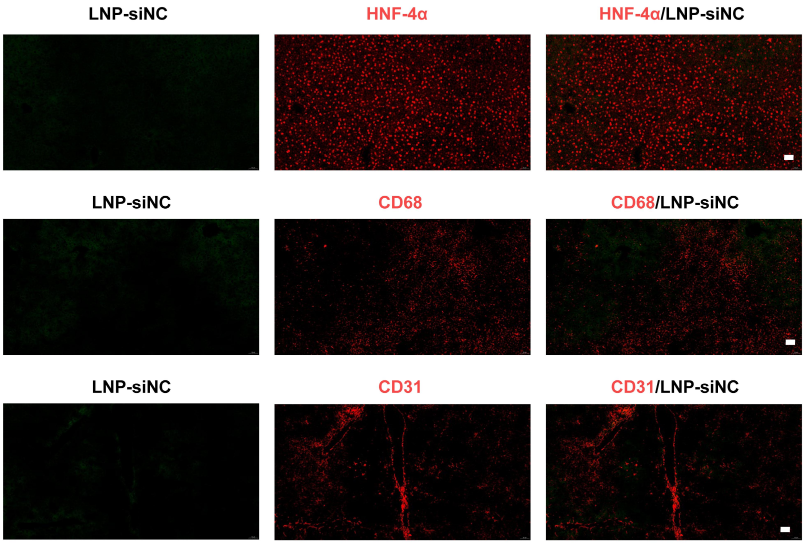

Fig. S6. Colocalization of HNF-4α/CD68/CD31 and nanoparticle without fluorescent labeling in the liver of mice (Scale bar = 50 μm).

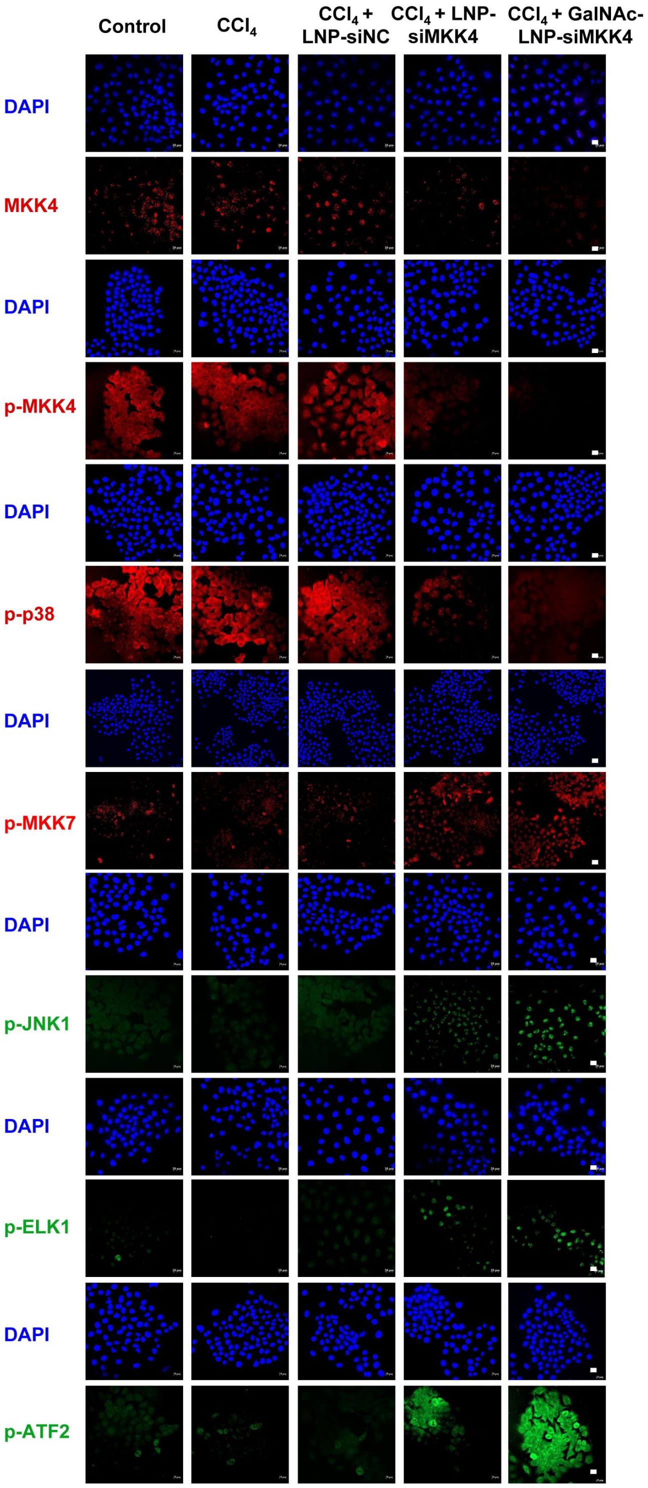

Fig. S7. Immunofluorescence analysis of MKK4, p-MKK4, p-p38, p-MKK7, p-JNK1, p-ELK1, and p-ATF2 in AML-12 cells after different treatments (Scale bar = 20 μm).

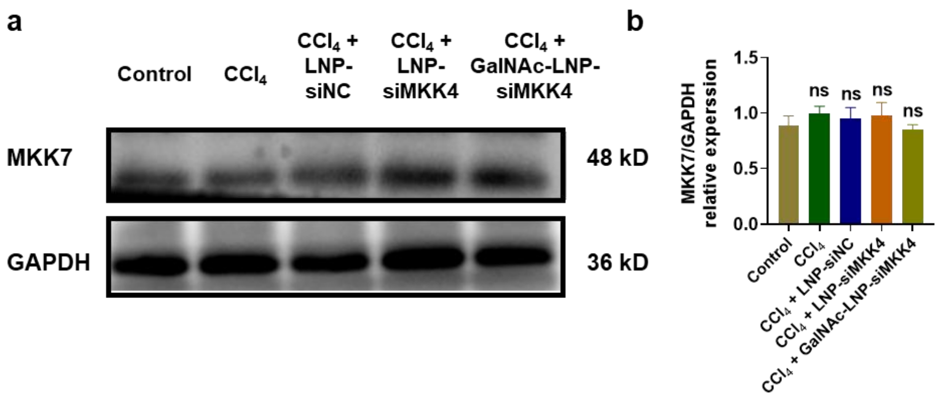

Fig. S8. Western Blot of MKK7 in AML-12 cells after different treatments. (a) Protein expression of MKK7. (b) Quantitative analysis on the protein expression of MKK7. mean ± SD, $n = 3$, ns (not significant, $p > 0.05$).

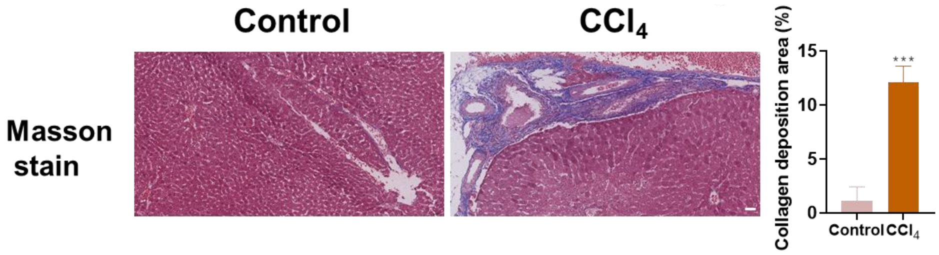

Fig. S9. Masson's trichrome staining of liver sections and quantification of collagen area percentage in healthy mice (Control) and mice after 4-week CCl_4 treatment (Scale bar = 50 μm). mean ± SD, $n = 6$, *** $p < 0.001$, compared to control group.

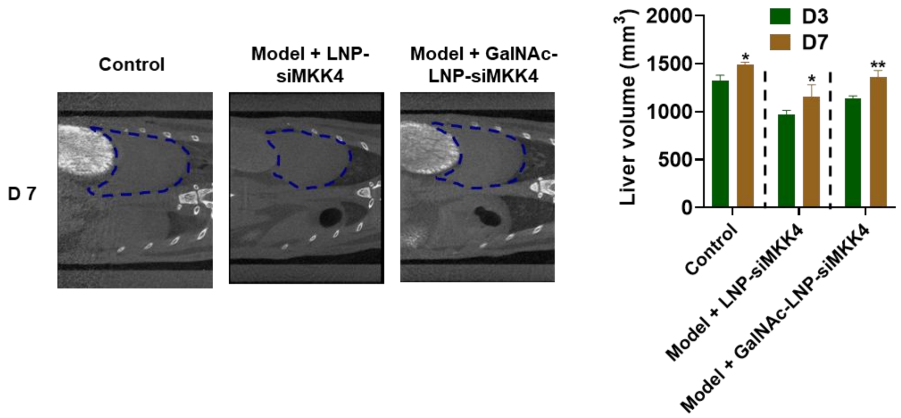

Fig S10. CT images of the liver on the 7th day and liver volume calculated based on quantitative segmentation of CT data. mean ± SD, $n = 6$. * $p < 0.05$ and ** $p < 0.01$, compared to 3rd day (D3).

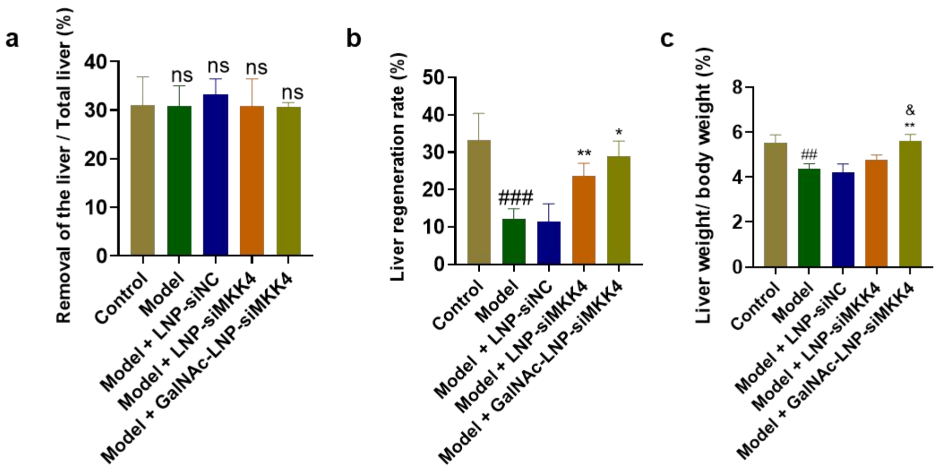

Fig. S11. Liver regeneration evaluation *in vivo.* (a) Liver resection to total liver ratio in each treatment group (mean ± SD; n = 3). (b) The liver regeneration ratio after different treatments (mean ± SD, n = 3), $^{\#\#\#}$ $p < 0.001$, compared to control group, ** $p < 0.01$ and *** $p < 0.001$, compared to model group. (c) The liver to body weight rate after different treatments (mean ± SD, n = 3), $^{\#\#\#}$ $p < 0.001$, compared to control group, ** $p < 0.01$, compared to model group, $^{\&}$ $p < 0.05$, compared to LNP-siMKK4 group.

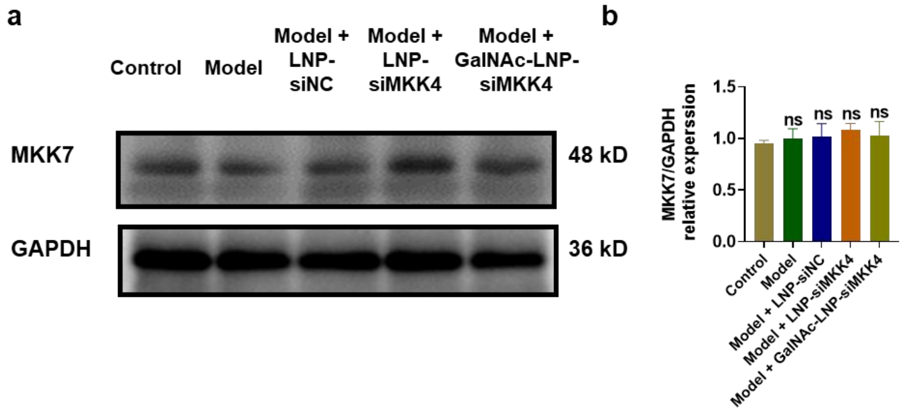

Fig. S12. Western Blot of MKK7 on acute-on-chronic liver failure of mice after different treatments. (a) The expression of MKK7 in liver tissues. (b) Quantitative analysis of MKK7 in liver tissue. mean ± SD, n = 6, ns (not significant, $p > 0.05$).

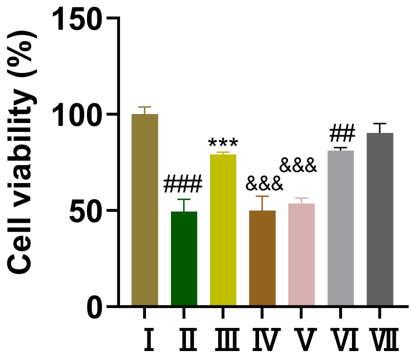

Fig. S13. The proliferative effect after different treatments. mean ± SD, $n = 6$, ## $p < 0.01$ and ### $p < 0.001$, II, VI and VII compared to I, *** $p < 0.001$, III compared to II, &&& $p < 0.001$, IV and V compared to III. I: control group, II: CCl_4 group, III: CCl_4 + GalNAc-LNP-siMKK4 group; IV: CCl_4 + GalNAc-LNP-siMKK4 group + Dehydrocorydaline chloride; V: CCl_4 + GalNAc-LNP-siMKK4 group + SP600125, VI: Dehydrocorydaline chloride (p38 agonist), VII: SP600125 (JNK1 inhibitor).

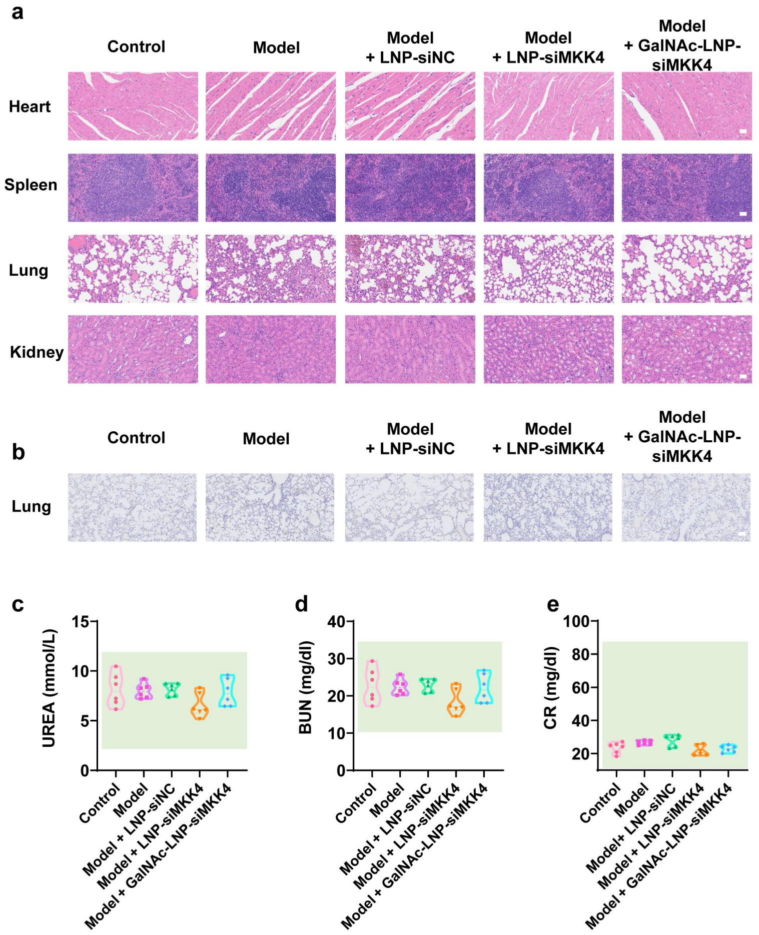

Fig. S14. The potential impact on major organs during the treatment period. (a) H&E staining of heart, spleen, lung, and kidney tissues (Scale bar = 50 μm). $n = 6$. (b) Immunohistochemical staining of CD163 in lung tissue (Scale bar = 50 μm). $n = 6$. (c-e) The concentrations of UREA, BUN, and CR in the serum. $n = 6$. Light-green area: the normal physiological range.

Table S1. The sequences of primers for quantitative real time polymerase chain reaction (RT-qPCR)

Genes	Description	Sequences of primers (5’ – 3’)
GADPH	*GADPH-F*	AATCGACAGCACGGTTTACTC
	GADPH-R	GCAGTGAAATCCCAGTGTTGTT
MKK4 (Map2k4-mouse-1158)	*MKK4-F*	AGGGCATCTTGGGCTACAC
	MKK4-R	TGGTCCAGGGTTTCTTACTCC

Table S2. The sequences of gene used in the experiments

Gene name	Description	Sequences (5’ – 3’)
siMKK4-1	*F*	GGACAGAAGUGGAAAUAUATT
	R	UAUAUUUCCACUUCUGUCCTT
siMKK4-2	*F*	CGCAUGCUAUGUUUGUAAATT
	R	UUUACAAACAUAGCAUGCGTT
siMKK4-3	*F*	CGGAAGAGAUCUUAGGCAATT
	R	UUGCCUAAGAUCUCUUCCGTT
siNC	*F*	UUCUCCGAACGUGUCACGUTT
	R	ACGUGACACGUUCGGAGAATT
siNC-FAM	*F*	FAM-UUCUCCGAACGUGUCACGUTT
	R	ACGUGACACGUUCGGAGAATT

Table S3. Survival analysis of ACLF mice after different treatment

*P-values adjusted for multiple comparisons using the Bonferroni method.

Comparison (A vs. B)	Hazard Ratio (HR)	95% Confidence Interval (CI)	Adjusted *P-value
Control vs. Model	0.027	0.002 - 0.343	< 0.05
Model + LNP-siNC vs. Model	1.095	0.193 - 6.228	ns
Model + LNP-siMKK4 vs. Model	0.027	0.002 - 0.343	< 0.05
Model + GalNAc-LNP-siMKK4 vs. Model	0.027	0.002 - 0.343	< 0.05
Model + LNP-siMKK4 vs. Model + GalNAc-LNP-siMKK4	Undefined	Undefined	ns
